# Enhancing credit card fraud detection with a hybrid approach using machine and deep learning

**DOI:** 10.1038/s41598-026-42891-4

**Published:** 2026-03-27

**Authors:** Nagwa Gamal, Eman M. G. Younis, Waleed M. Makram

**Affiliations:** 1 Faculty of Computers and Information, Minia University, Minia, Egypt; 2 Faculty of Computers and Artificial Intelligence, Minia National University, Minia, Egypt

**Keywords:** Deep learning, Machine learning, Ensemble learning, Stacking ensemble, Imbalanced data, SMOTE, Fraud detection, Engineering, Mathematics and computing

## Abstract

Credit card fraud is an important concern for banks, financial institutions and consumers, resulting in substantial financial losses annually. Traditional fraud detection systems are based on predefined rules, but as fraudsters develop more sophisticated techniques, these methods become less effective. Machine learning (ML) and deep learning (DL) offer powerful solutions to enhance the accuracy and efficiency of credit card fraud detection. However, a major challenge in credit card fraud detection is the highly imbalanced nature of transaction data, where fraudulent transactions are rare compared to legitimate ones. To address data imbalance, techniques such as Synthetic Minority Over-sampling Technique (SMOTE) and Mahalanobis distance Synthetic Minority Oversampling Technique–Edited Nearest Neighbors (SMOTE-ENN) hybrid sampling are applied to balance the dataset and improve model performance. The study evaluates various ML models and deep learning models for fraud detection and further evaluate learning dynamics, computational cost, and interpretability through SHAP (SHapley Additive exPlanations) and LIME (Local Interpretable Model-agnostic Explanations). Comprehensive error analysis confirms the robustness and transparency of the proposed approach. This study evaluated 37 models, and the two proposed stacking ensemble approaches showed significant advancements. The first proposed model effectively combines various algorithms: Extra Trees (ET), Convolutional Neural Networks (CNN), Long Short-Term Memory (LSTM), and eXtreme Gradient Boosting(XGBoost) as a meta-learner and the second proposed stacking ensemble approach integrates ET, Adaptive Boosting(AdaBoost) with Extra Trees as base, AdaBoost with Random Forest as base, XGBoost as meta-learner to maximize performance. This research highlights the importance of combining machine learning, deep learning, and data balancing techniques to improve credit card fraud detection. The proposed stacking ensemble approaches achieved exceptional results, with accuracy, precision, recall, F1-score, and Area Under the Curve (AUC) reaching 1.0, 0.9999, 1.0, 1.0 and 1.0, respectively. Experimental results indicate that ensemble learning techniques like Categorical Boosting (CatBoost) and XGBoost outperform traditional models, while deep learning methods, especially Feedforward Neural Network (FFNN), ANN (Artificial Neural Network) and Multilayer Perceptron(MLP) demonstrate strong performance in detecting fraud patterns.

## Introduction

Fraud is generally defined as a deceptive act intended for financial gain. In the context of credit card transactions, fraud can be categorized into internal or external. Internal fraud occurs when the fraudster assumes a false identity with the collusion of the cardholder and the financial institution. In contrast, external fraud involves unauthorized access to a credit card to extract money or make transactions through deceptive means^1^.

Financial fraud detection has emerged as a critical area of research due to the increasing reliance on digital financial transactions as well as the growing sophistication of fraud schemes. The rapid expansion of e-commerce, online banking, and cashless payment methods has created an urgent need for effective fraud detection systems. These systems are used to mitigate financial losses and protect consumers and institutions^2^. Fraudulent transactions, particularly in credit card payments, often involve unauthorized access through phishing, data breaches, and cyber scams, making traditional rule-based detection methods insufficient in handling modern fraud schemes^3,4^.

The financial impact of fraud is substantial and growing. The Nilson Report estimates that global credit card fraud losses reached $33.45 billion in 2022 and are projected to exceed $43 billion by 2028^5^. According to the FTC^6^, fraud reports in the United States reached 2.4 million cases in 2024, with losses of approximately $8.8 billion, marking a significant increase from 2023. These escalating losses underscore the need for advanced artificial intelligence-driven detection systems to strengthen security and mitigate economic risks.

Fraud can be mitigated through two primary approaches: prevention and detection. Prevention serves as a protective barrier, stopping fraudulent activities before they occur. Detection, on the other hand, comes into play when preventive measures have failed, identifying and responding to fraudulent actions after they have taken place^7^. While prevention aims to block fraud before it happens, this study focuses on detection identifying fraudulent activities, analyzing patterns, and ensuring timely intervention to minimize damage.

Machine learning (ML) and deep learning (DL) have emerged as powerful tools in financial fraud detection due to their ability to learn complex patterns from large datasets^8^. They offer the advantage of learning from historical data to make accurate predictions and adapt to evolving fraud tactics^9,10^. Supervised learning models, such as logistic regression, decision trees, support vector machines (SVM), and random forests, have been widely used to classify transactions as fraudulent or legitimate^1,9^. Traditional methods have been complemented by more advanced algorithms such as gradient boosting (e.g., XGBoost, LightGBM, and CatBoost), and deep learning architectures including convolutional and recurrent neural networks^3,11^.

However, a major challenge in fraud detection is the high class imbalance present in financial datasets, where fraudulent transactions typically constitute a tiny fraction of overall transactions^12^. To address this issue, various resampling techniques such as SMOTE, ADASYN, and hybrid approaches^10,13,14^, as well as generative models like GANs^12,15^, have been developed to balance datasets and improve model performance.

Additional techniques have proven valuable in enhancing fraud detection. Feature engineering helps extract meaningful transaction patterns, while autoencoders and representation learning improve feature selection and classification accuracy^8^. Recurrent Neural Networks (RNNs), including Long Short-Term Memory (LSTM) and Gated Recurrent Units (GRUs), have been employed to capture temporal transaction patterns^8^. Ensemble learning approaches, which combine multiple classifiers through strategies such as majority voting and weighted classifiers, have demonstrated significant improvements in fraud detection performance^3,16^.

### Contributions

This work makes the following contributions to the field of credit card fraud detection:

1. Robust handling of class imbalance: We address the extreme skew in fraud datasets by applying advanced resampling strategies, including Mahalanobis SMOTE-ENN and SMOTE, ensuring fairer and more reliable model training.
2. Extensive benchmarking: We systematically evaluate 37 baseline models alongside two newly proposed ensemble models, providing one of the most comprehensive comparative studies in this domain.
3. Learning dynamics and efficiency: We analyze learning curves and computational costs, offering insights into scalability and practical deployment feasibility for financial institutions.
4. Model interpretability: We integrate explainable AI (XAI) techniques (SHAP and LIME), allowing both local and global interpretability of model decisions, thus enhancing trust and transparency.
5. Error analysis: We perform a detailed error analysis to understand failure cases and provide directions for further model refinement.

Together, these contributions not only advance predictive performance but also improve transparency, efficiency, and trustworthiness of fraud detection systems.

The rest of the paper is organized as follows. Section II presents related work. Section III describes the proposed approach for detecting credit card fraud. The experiments conducted and their analyses are presented in Section IV. Section V provides the computational cost analysis. Section VI presents the model generalization assessment for the proposed ensemble models. Section VII discusses the explainable AI (XAI) analysis. Section VIII contains the error analysis and model comparison. Section IX outlines the research limitations. Finally, Section X includes the conclusions of the paper, as well as recommendations for future research.

## Related work

Various ML and DL techniques have been used in experimental research to detect and predict fraudulent transactions. Many of these studies utilize the European Credit Card Dataset containing 284,807 transactions with 492 fraudulent cases (0.172% fraud rate), highlighting the severe class imbalance challenge inherent in fraud detection. Ensemble and Hybrid Deep Learning Approaches: Multiple studies have demonstrated the effectiveness of ensemble methods combining diverse architectures. An integrated approach combining CNNs, LSTM, and Transformers with XGBoost as a meta-learner achieved 0.972 AUC-ROC on the European dataset and 0.920 on the Taiwan dataset^17^. A combination of Gradient Boosting, Random Forest, and Logistic Regression with hyperparameter tuning achieved 99.59% accuracy^18^. An ensemble combining RUS, two-stage thresholding, and SMOTE achieved 99.07% accuracy with SMOTE enhancement^5^. Research investigating ensemble techniques integrating SVM, KNN, Random Forest, Bagging, and Boosting classifiers found that RF and Boosting achieved 100% accuracy on training samples and 99.98% on testing data using SMOTE^19^. Advanced Sampling and Generative Techniques: Addressing class imbalance remains critical in fraud detection research. R-GAN incorporating spectral normalization and similarity measure loss outperformed traditional oversampling methods with 99.5% accuracy^20^. ADASYN with RFECV feature selection achieved 99.98% accuracy, with XGBoost showing the highest MCC of 0.9994^14^. A hybrid approach combining Tomek links undersampling with BCBSMOTE oversampling on the PaySim dataset achieved 85.20% F1-score^13^. CB-CHL-LightGBM and OS-CHL-LightGBM integrating cost-sensitive learning improved F2-scores from 0.77 to 0.86 across three datasets^21^. Autoencoder and Anomaly Detection Approaches: Autoencoders have proven effective for anomaly detection in fraud detection. CNN-based VAE models for latent space representation achieved 0.92 F1-score and outperformed traditional resampling with machine learning models^22^. GNNs with Lambda Architecture and Autoencoders on Pakistani bank datasets achieved approximately 78% accuracy, with the Gated Temporal Attention Network (GTAN) proving particularly robust in low-label settings^23^. Federated Learning and Privacy-Preserving Approaches: Recent research has emphasized privacy-preserving fraud detection through federated learning. A Structured Data Transformer combined with federated learning and momentum updates achieved AUC-ROC values of 0.982 and 0.994 on two datasets^24^. FedFusion for adaptive model fusion across distributed clients with feature discrepancies achieved detection rates of 99.74%, 99.70%, and 96.61% across three clients while maintaining privacy^25^. Recent Advanced Methods: An anomaly detection framework based on multi-view heterogeneous graph neural networks was proposed by Berkmans et al.  ^26^. Their pipeline incorporated FSMOTE for class balancing, MFKF for missing values, a metaheuristic-based feature selection method, and QAHA for hyperparameter tuning, delivering consistently higher accuracy, precision, and F1-scores than existing methods. A deep learning ensemble combining Kolmogorov–Arnold Networks with dynamic oversampling and ensemble feature selection was introduced by Akouhar et al.^27^, applying GAN-based oversampling with adaptive sampling rates and metaheuristic-driven feature selection. Experiments on three benchmark datasets showed it outperformed conventional deep learning approaches. The Balanced Variational AutoEncoder with Attention (Bal-VAE-Attention) was developed by Shi et al.  ^28^, integrating an imbalance-aware loss function, LightGBM-based feature selection, and multi-head attention to capture complex fraudulent patterns. A stacking ensemble combining SVM, KNN, and a PSO-optimized Extreme Learning Machine was proposed by Gupta et al.  ^29^, enhanced with SMOTE-ENN preprocessing, autoencoder-based dimensionality reduction, and TOPSIS feature selection, achieving 99.95% accuracy and 99.97% recall. XGBoost with Bayesian optimization was applied by Tayebi et al.  ^30^, coupling Bayesian hyperparameter tuning with SMOTE and Random Under-Sampling, delivering high accuracy (up to 0.9996) and AUC close to 0.99 while providing interpretable results.

To offer a clearer comparison, the related work is summarized in Table 1.

**Table 1:** Summary of related work.

Refs.	Dataset	Methods	Proposed model	Results of the proposed model
^17^	European Credit Card Dataset (284,807 transactions, 492 fraud cases); Taiwan Credit Card Dataset (30,000 transactions, 1,000 fraud cases)	—	CNN, LSTM, Transformers, XGBoost as final decision-maker (Stacking Ensemble)	Sensitivity: 0.961 Specificity: 0.999 AUC-ROC: 0.972 (European dataset). Sensitivity: 0.918 Specificity: 0.942 AUC: 0.920 (Taiwan dataset)
^24^	Dataset 1: European Credit Card Dataset, Dataset 2: Simulated dataset	—	Federated Structured Data Transformer (SDT)	AUC-PR: 0.884 , 0.892 AUC-ROC: 0.963 , 0.998 with federated learning
^5^	European Credit Card Dataset	Random Under-Sampling (RUS), Two-Stage Thresholding (TST), SMOTE	LR, KNN, SVM, DT and the proposed ensemble model	Accuracy: 99.1% (SMOTE) Precision: 99.9% Recall: 99.1% F1-score: 99.5%
^20^	European Credit Card Dataset	Random Over-Sampling, SMOTE, ADASYN, Borderline-SMOTE, WGAN, WGANGP, SNGAN	Regularized Generative Adversarial Network (R-GAN)	Accuracy: 99.5% Precision: 100% Recall: 99.0% F1-score: 99.5%
^14^	European Credit Card Dataset	Adaptive Synthetic Minority Oversampling (ADASYN), Recursive Feature Elimination with Cross-Validation (RFECV)	LGBM, RF, DT, LR ,Proposed XGB	XGB: MCC 0.999 Accuracy: 99.9% LGBM: MCC improved from 0.339 to 0.998
^22^	European Credit Card Fraud Dataset	SMOTE, Under-sampling, Over-sampling	CNN-based VAE (VAE-normal and VAE-anomaly)	F1-score: 0.92 (VAE-normal), 0.91 (VAE-anomaly); Outperformed traditional ML models
^23^	Pakistani Banks (Meezan Bank & UBL) (284,807 transactions, 492 fraud cases)	Min-Max Scaling	Graph Neural Networks (GNNs), Autoencoders	GNN Accuracy: 78.1% (Meezan), 78% (UBL). Autoencoder Accuracy: 77.1% (Meezan), 79% (UBL)
^19^	European Credit Card Dataset	Under-sampling, SMOTE	SVM, KNN, RF, Bagging, Boosting, Proposed Model (P_M_2)	SMOTE: Accuracy 99.98%, Precision, Recall, and F1-score highest for P_M_2
^25^	Dataset 1: European Credit Card Transactions; Dataset 2: Kaggle Dataset (8 features); Dataset 3: Synthetic dataset (300M transactions)	Federated learning techniques for data heterogeneity	CNN, LSTM, MLP (MLP as the global model)	Detection Rate: 99.7%, 99.7%, 96.6% (Clients 1, 2, and 3); Accuracy: 99.9%, 99.5%, 99.1%
^13^	PaySim Dataset (6M synthetic credit card transactions)	Combines Tomek Links (undersampling) and BIRCH Clustering Borderline SMOTE (BCBSMOTE)	Random Forest (RF)	Accuracy: 99.9% F1-score: 85.2% Precision: 81.3% Recall: 89.5% AUPRC: 72.7%
^21^	Three datasets including European Credit Card Transactions	Class Balancing, Oversampling, Cost-sensitive Learning	CB-CHL-LightGBM, OS-CHL-LightGBM	F2-score: 0.77 to 0.83 Cost savings improved from 0.65 to 0.76
^18^	European Credit Card Dataset	Under-sampling, SMOTE	RF, Logistic Regression, Voting Classifiers and Gradient Boosting achieved the best performance	Accuracy: 99.6% Precision: 97.3% Recall: 98.5%, F1-score: 98.9%
^26^	Credit Card Fraud (29k)	FSMOTE (balancing), MFKF (missing data), SAA (feature selection)	AD-OCCD-MHGNN (MHGNN + QAHA)	Accuracy 99.5% Precision 98.9% Recall 98.9% F1 98.7%
^27^	Three benchmark fraud datasets	SMOTE, GAN oversampling, Ensemble feature selection	KAN + GAN-based ensemble	Accuracy 99.9% Precision 98.3% Recall 94.1% F1 96.1%
^28^	Two open-source CCF datasets	Bal-VAE oversampling, LightGBM feature selection	Bal-VAE-Attention	Recall 99.9% Precision 99.9% AUC-ROC 99.9% AUC-PR 99.9%
^29^	Standard CCF datasets	SMOTE-ENN, Autoencoder dim. reduction, TOPSIS feature selection	Stacking (SVM, KNN, PSO-ELM)	Accuracy 99.9% Precision 99.9% Recall 99.9% AUC = 1.00
^30^	Two Kaggle CCF datasets	SMOTE, Random Undersampling, CV, Bayesian optimization	XGBoost (BO-optimized)	Data 1 (SMOTE): Accuracy 99.9% Precision 94% Recall 87.4% F1 0.87.4% AUC 98.8Data 2 (RUS): Accuracy 83.3% Precision 82.9% Recall 83.8% F1 83.4% AUC 90.9%

## The proposed approach for detecting credit card fraud

### Dataset description

**Fig. 1:**
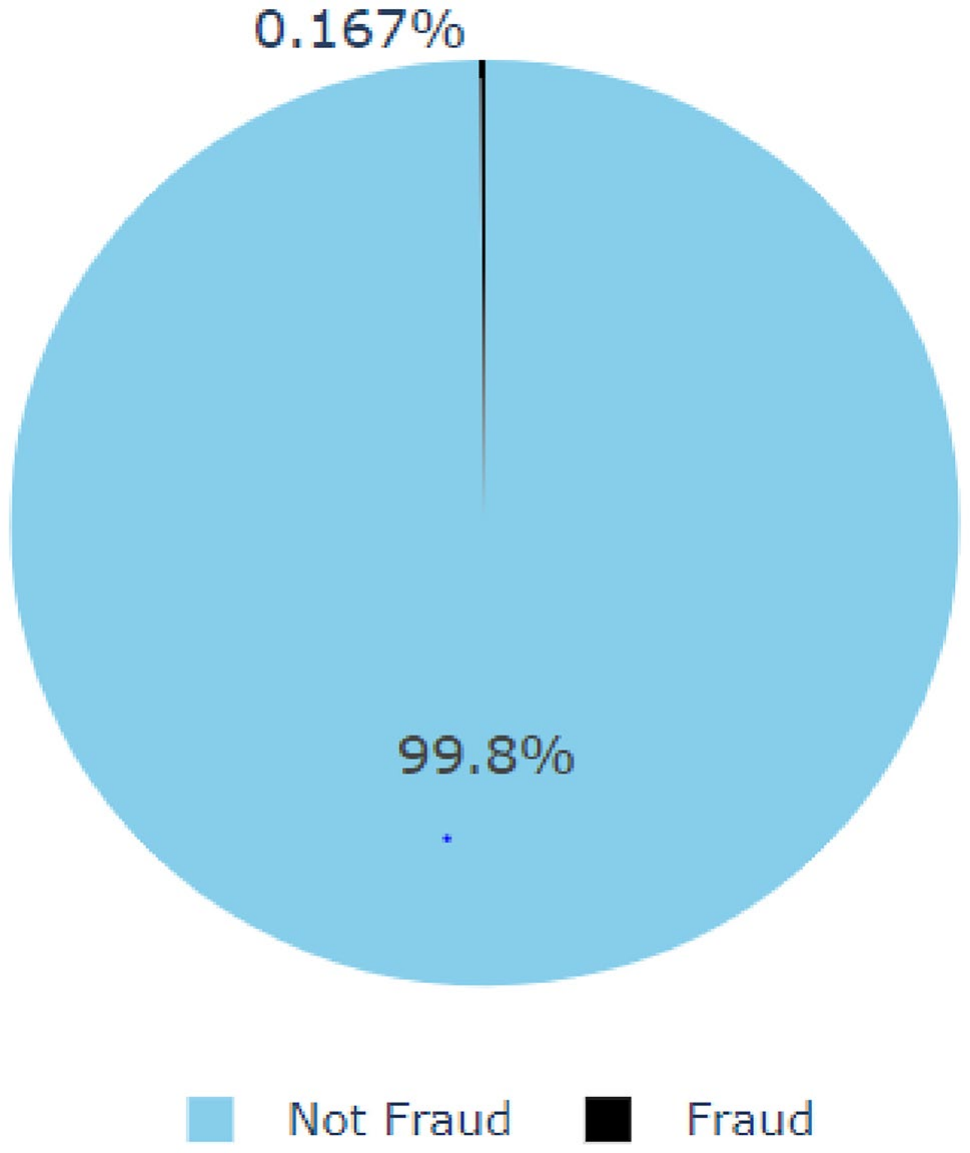
Percentage between Frauds and genuine transactions.

This study utilizes a real-world dataset, known as “creditcard,”^31^ to ensure the proposed algorithm’s practical applicability. The data set contains 284,807 transaction records collected over two days of credit card use in September 2013. Of these, 492 transactions are fraudulent, accounting for only 0.172% of all transactions, which highlights the highly imbalanced nature of the dataset (as seen in Fig. 1).

The dataset is publicly available on Kaggle and can be accessed at Credit Card Dataset. It consists entirely of numerical features generated using Principal Component Analysis (PCA). The original features and detailed background information were omitted to maintain confidentiality and privacy. It includes 28 principal components (labeled V1 to V28) derived through PCA, along with two untransformed features: Time, representing the elapsed time (in seconds) since the first transaction, and Amount, indicating the monetary value of each transaction. The target variable, Class, is binary, with 1 representing fraudulent transactions and 0 indicating legitimate ones. A summary of the features is provided in Table  2 .

**Table 2:** Features of the credit card fraud dataset that is used in this paper.

Variable name	Description	Type
V1, V2, ..., V28	Transaction feature after PCA transformation	Integer
Time	Seconds elapsed between each transaction with the first transaction	Integer
Amount	Transaction value	Integer
Class	Legitimate or Fraudulent	0 or 1

### Data pre-processing

**Fig. 2:**
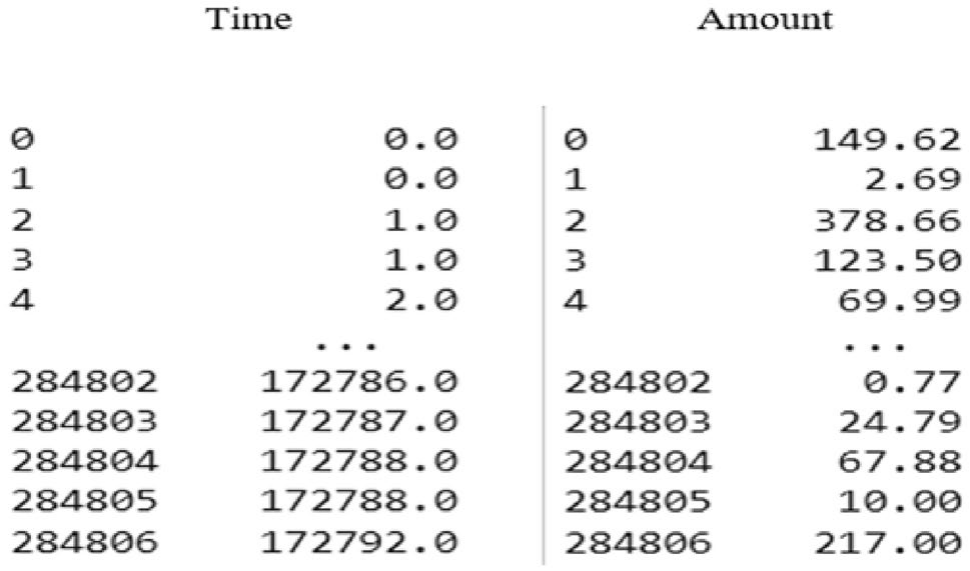
Values of the amount and time columns in the dataset.

**Fig. 3:**
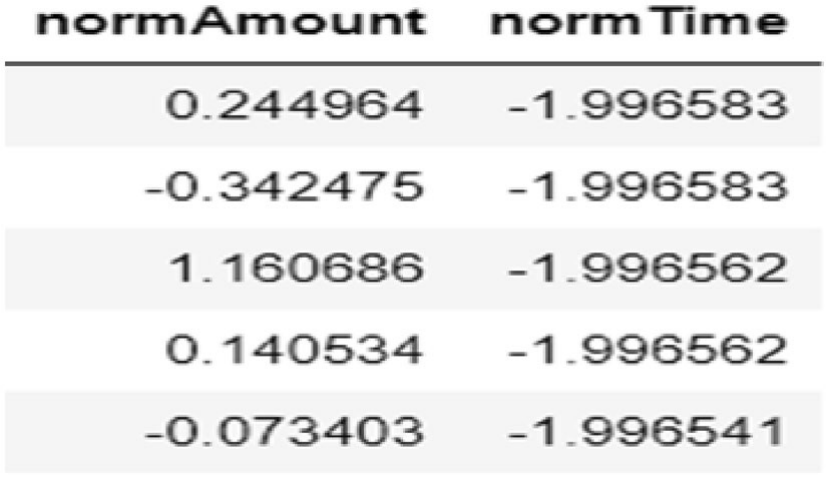
Normalized values of amount and time.

The credit card fraud dataset had already been normalized for features V1 to V28, with no anomalies or missing values, so no additional processing was required for those aspects. However, the features Amount and Time showed a significant difference in value distributions (as seen in Fig. 2). Without processing, this could skew the system’s assessment of the importance of these features due to their scale differences. To address this, it was necessary to standardize the data for Amount and Time. A new field, normAmount, was created to store the standardized values for the Amount feature, while the Time values were replaced with normTime to store the standardized equivalents (as shown in Fig. 3).

### Machine learning and deep learning classifiers

Table  3 presents the 37 classification models employed in this study and the two proposed stacking ensemble models, categorized into Traditional Machine Learning, Boosting Algorithms, Bagging Algorithms, Neural Networks, Recurrent Neural Networks, and Ensemble Methods. These models are analyzed and compared to assess their impact on credit card fraud detection.

**Table 3:** Classification models used for credit card fraud detection.

Category	Models
Traditional machine learning	Naive Bayes, Decision Tree, Logistic Regression, Random Forest (RF), K-Nearest Neighbors (KNN), Linear Discriminant Analysis, Bernoulli NB, Ridge Classifier, Extra Trees Classifier, Dummy Classifier, Quadratic Discriminant Analysis, SGD Classifier
Boosting algorithms	Gradient Boosting, XGBoost, LightGBM, CatBoost, AdaBoost with Decision Tree base, AdaBoost with Extra Trees base, AdaBoost with Logistic Regression base, AdaBoost with Random Forest base, AdaBoost with Ridge Classifier base
Bagging algorithms	Bagging Classifier with Decision Trees
Neural networks	Neural Network, Artificial Neural Network (ANN), Feedforward Neural Network (FFNN), Multilayer Perceptron (MLP), Convolutional Neural Network (CNN)
Recurrent neural networks	Recurrent Neural Network (RNN), Long Short-Term Memory (LSTM), Gated Recurrent Unit (GRU), Bidirectional LSTM (BiLSTM), Bidirectional GRU (BiGRU), BiLSTM with Maxpooling, BiGRU with Maxpooling
Ensemble methods	Stacking Classifier (RF, CNN, LSTM, XGBoost, LogisticRegression as meta-learner), Stacking Classifier (RF, CNN, LSTM, XGBoost as meta-learner), Stacking Classifier (CNN, LSTM, Transformer Base Learners, XGBoost as meta-learner)
Proposed models	Stacking Classifier (ET, CNN, LSTM, XGBoost as meta-learner), StackingClassifier (ET, AdaBoostClassifier with ExtraTreesClassifier as base, AdaBoostClassifier with RandomForestClassifier as base, XGBoost as meta-learner)

**Fig. 4:**
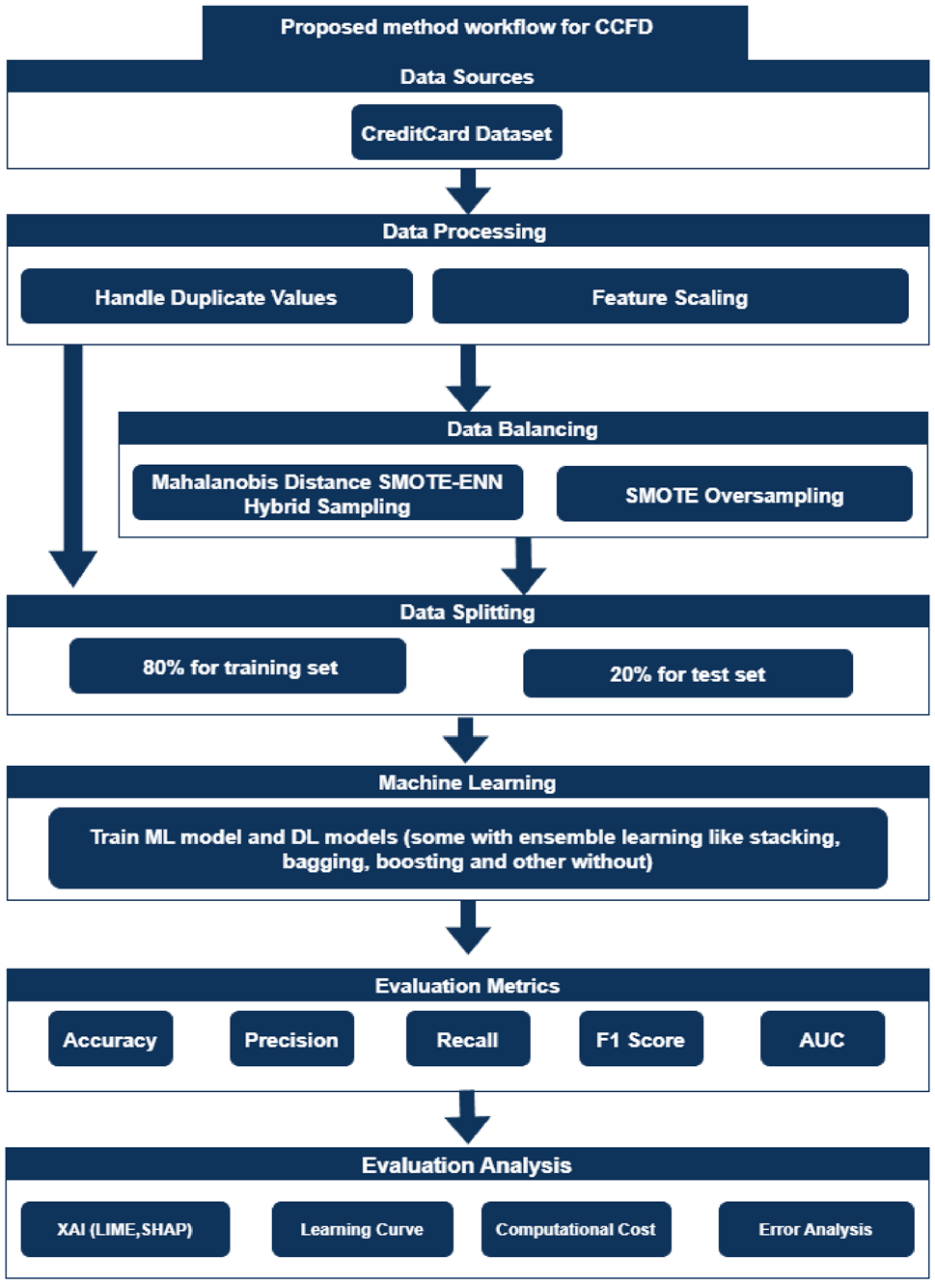
The proposed approach for credit card fraud detection.

This study presents a credit card fraud detection approach (as seen in Fig. 4) consisting of two main parts. First, all classification models are trained individually using the original, imbalanced dataset to maintain its natural distribution. Second, the dataset is balanced using two methods SMOTE and a hybrid model that combines Mahalanobis distance and SMOTE-ENN. The balanced dataset is then used to retrain the same 37 classification models along with the two proposed models. These models are evaluated using metrics such as accuracy, precision, recall, F1-score, and AUC diagrams. The steps can be summarized as follows:

- Data Preprocessing.
- Train 39 Classification Models and Evaluate Using Metrics.
- Apply SMOTE oversampling and Mahalanobis Distance SMOTE-ENN Hybrid Sampling Methods.
- Retrain the Models on the Balanced Data and Reevaluate.
- Compare Performance Metrics Before and After Balancing.

This experimental approach assesses the impact of balancing techniques and enhanced training data on credit card fraud detection. It demonstrates how these methods can improve the performance, effectiveness, and robustness of classification models, offering a practical and reliable solution for real-world applications.

### Ensemble learning

Ensemble learning is a machine learning technique that combines multiple models to improve decision-making in supervised learning tasks. This approach addresses common issues in individual models, such as high bias, variability, and limited accuracy^32^. Instead of relying on a single model, ensemble learning leverages the strengths of multiple classifiers to achieve better performance. It is generally categorized into three main types: bagging, boosting, and stacking. Bagging (bootstrap aggregating) trains several learners independently on bootstrap samples drawn from the training data and aggregates their predictions through voting or averaging, thereby reducing variance and increasing stability, particularly for tree-based models. In contrast, boosting constructs models sequentially, with each new learner emphasizing instances that were misclassified by its predecessors, which helps reduce bias and often yields strong performance, as demonstrated by algorithms such as AdaBoost and XGBoost^33,34^. Stacking involves combining multiple base models, known as base learners, and using a higher-level model, called a meta-learner, to refine their predictions. This process consists of two layers: the base layer, where multiple models generate predictions based on input data, and the meta layer, which integrates these predictions to enhance overall accuracy. By combining different models, stacking improves predictive performance and reduces the likelihood of errors^34^.

### Data imbalance handling methods

This section covers the approaches used to address data imbalance in the study, including SMOTE oversampling and the hybrid SMOTE-ENN method based on Mahalanobis distance.

#### SMOTE

SMOTE is an algorithm designed to address class imbalance in datasets by generating synthetic samples to balance the class distribution^35^. It works by selecting samples from the minority class and identifying several of their nearest neighbors. New samples are then created through random interpolation between the selected samples and their neighbors. By adjusting the interpolation ratio, SMOTE can control the impact of the synthetic samples on the training set^36,37^.

#### Mahalanobis distance SMOTE-ENN hybrid sampling

The Synthetic Minority Oversampling Technique (SMOTE) is commonly used to address class imbalance by creating artificial examples for the minority class. In contrast, undersampling methods such as Edited Nearest Neighbor (ENN) reduce the number of instances from the majority class to achieve balance. However, this approach can result in the removal of valuable data and tends to be less effective when there is a large disparity between the majority and minority classes, as seen in the credit card dataset under study. Additionally, while oversampling helps balance the data, it may lead to overfitting by duplicating existing samples^16^. To tackle these issues, this study uses the SMOTE-ENN method, a hybrid resampling technique that combines oversampling (via SMOTE) and undersampling (via ENN) to create a balanced dataset. SMOTE generates new synthetic samples for the minority class, while ENN removes overlapping and noisy samples from the majority class using a neighborhood cleaning rule. This rule eliminates instances that differ from at least two out of their three nearest neighbors^16,38^. For this study’s credit card fraud dataset, the data is not only imbalanced but also multidimensional and includes anomalies. To handle this complexity, the Mahalanobis distance is used as the distance metric for SMOTE-ENN. The Mahalanobis distance takes into account the correlation between features and effectively measures the distance between samples in high-dimensional spaces. It is robust against outliers, reducing their influence on distance calculations^39^. By incorporating the Mahalanobis distance, the SMOTE-ENN technique better captures feature correlations, adapts to multidimensional datasets, and improves classification performance. This approach effectively handles imbalanced datasets, making it a suitable solution for credit card fraud detection^39^.

### Evaluation metrics

- Confusion Matrix : A confusion matrix (as shown in Table  4) is an n$\times$n table that summarizes how a classification model’s predictions align with the actual outcomes. In the case of a 2$\times$2 matrix, it provides details on true positives (TP), true negatives (TN), false positives (FP), and false negatives (FN)^40^.

**Table 4:** Confusion matrix.

Actual values	Positive	Negative
Predicted values		
Positive	TP (True Positive)	FP (False Positive)
Negative	FN (False Negative)	TN (True Negative)
- Accuracy: The accuracy rate represents the proportion of correct predictions made by a classifier out of the total predictions on a test set. It is essentially the inverse of the error rate, with both metrics offering similar insights into the classifier’s performance^40,41^.
  1 $$\begin{aligned} \text {Accuracy} = \frac{TP + TN}{TP + TN + FP + FN} \end{aligned}$$
- Precision : It is the ratio of true positives to the total number of positive predictions made by the classifier, including both true positives and false positives that were mistakenly identified as positives^40,42^.
  2 $$\begin{aligned} \text {Precision} = \frac{TP}{TP + FP} \end{aligned}$$
- Recall : Also known as sensitivity or the true positive rate, it measures a model’s ability to correctly identify true positive cases^40^.
  3 $$\begin{aligned} \text {Recall} = \frac{TP}{TP + FN} \end{aligned}$$
- The F1 score : Also called the F-measure, it represents the harmonic mean of precision and recall, providing a balanced evaluation of a classification model’s performance^41,42^.
  4 $$\begin{aligned} \text {F1-Score} = 2 \times \frac{\text {Precision} \times \text {Recall}}{\text {Precision} + \text {Recall}} \end{aligned}$$
- Receiver Operating Characteristic (ROC) Curve/Area Under the ROC Curve: The ROC curve is a visual representation of a classification model’s performance across various thresholds. It plots the True Positive Rate (TPR) against the False Positive Rate (FPR) at each threshold^40,43^.
  5 $$\begin{aligned} \text {TPR (Recall)} = \frac{TP}{TP + FN} \end{aligned}$$
  6 $$\begin{aligned} \text {FPR} = \frac{FP}{FP + TN} \end{aligned}$$

### Experimental setup and reproducibility

To ensure full reproducibility of our experimental results, all implementation details are documented below.

#### Software environment

All experiments were conducted using Python 3.11.5 with the following libraries: TensorFlow 2.18.0, scikit-learn 1.2.2, XGBoost 2.0.3, NumPy 1.24.0, and Pandas 1.5.3.

#### Hardware configuration

All experiments were conducted on a VMware virtual machine with Intel Xeon Gold 6338 CPU @ 2.00GHz, 64GB RAM, and 280GB HDD storage.

#### Hyperparameter selection rationale

Hyperparameters were selected based on established literature and preliminary experiments: Input shape (30, 1) reflects dataset structure; CNN filters (64, 128) and LSTM units (100, 50) follow recent practices for deep learning on tabular data; Extra Trees n_estimators=100 aligns with ensemble learning literature; AdaBoost/XGBoost parameters follow validated configurations; deep learning training (20 epochs, batch size 32, Adam optimizer) determined through convergence monitoring and standard practices^44,45^.

#### Model configuration

Complete hyperparameter specifications, random seed settings, and training configurations for both proposed models are documented in Tables 5 and 6.

**Table 5:** Hyperparameter configuration for proposed model 1: extra trees-CNN-LSTM stacking ensemble.

Model Component	Parameters	Training Config
CNN Model	Input Shape: (30, 1) Conv1D Layer 1: 64 filters, kernel size 3, ReLU activation MaxPooling1D Layer 1: pool size 2 Conv1D Layer 2: 128 filters, kernel size 3, ReLU activation MaxPooling1D Layer 2: pool size 2 Flatten Layer Dense Layer: 64 neurons, ReLU activation Output Layer: 1 neuron, sigmoid activation Optimizer: Adam (learning rate=0.001, $\beta _1$=0.9, $\beta _2$=0.999, $\epsilon$=1e-07) Loss Function: Binary Crossentropy Metrics: Accuracy	Epochs: 20 Batch Size: 32 Verbosity: 0
LSTM Model	Input Shape: (30, 1) LSTM Layer 1: 100 units, return_sequences=True LSTM Layer 2: 50 units Dense Layer: 64 neurons, ReLU activation Output Layer: 1 neuron, sigmoid activation Optimizer: Adam (learning rate=0.001, $\beta _1$=0.9, $\beta _2$=0.999, $\epsilon$=1e-07) Loss Function: Binary Crossentropy Metrics: Accuracy	Epochs: 20 Batch Size: 32 Verbosity: 0
Extra Trees Classifier	n_estimators: 100 criterion: gini max_depth: None min_samples_split: 2 min_samples_leaf: 1 max_features: sqrt bootstrap: False random_state: None n_jobs: None verbose: 0	Used as base learner in stacking ensemble
XGBoost Classifier (Meta-Learner)	objective: binary:logistic eval_metric: logloss use_label_encoder: False n_estimators: 100 (default) max_depth: 6 (default) learning_rate: 0.3 (default) subsample: 1.0 (default) colsample_bytree: 1.0 (default) random_state: None	Trained on meta-features from base learners
Stacking Classifier Configuration	Base Learners: Extra Trees, CNN, LSTM Final Estimator: XGBoost stack_method: auto passthrough: False n_jobs: None verbose: 0	Cross-validation strategy for generating meta-features

**Table 6:** Hyperparameter configuration for proposed model 2: ensemble boosting stacking.

Model component	Parameters	Training Config
Extra Trees Classifier	n_estimators: 100 criterion: gini max_depth: None min_samples_split: 2 min_samples_leaf: 1 max_features: sqrt bootstrap: False random_state: 42 n_jobs: None verbose: 0	Used as base learner in stacking ensemble
AdaBoost + Random Forest	Base Estimator: RandomForestClassifier - n_estimators: 10 - criterion: gini - max_features: sqrt - bootstrap: True algorithm: SAMME.R n_estimators: 50 learning_rate: 1.0 random_state: 42	Boosting iterations: 50
AdaBoost + Extra Trees	Base Estimator: ExtraTreesClassifier - n_estimators: 100 (default) - criterion: gini - max_features: sqrt - bootstrap: False algorithm: SAMME.R n_estimators: 50 learning_rate: 1.0 random_state: 42	Boosting iterations: 50
XGBoost Classifier (Meta-Learner)	objective: binary:logistic eval_metric: logloss use_label_encoder: False n_estimators: 100 (default) max_depth: 6 (default) learning_rate: 0.3 (default) subsample: 1.0 (default) colsample_bytree: 1.0 (default) random_state: None	Trained on meta-features from base learners
Stacking Classifier Configuration	Base Learners: Extra Trees, AdaBoost+RF, AdaBoost+ET Final Estimator: XGBoost stack_method: auto passthrough: False n_jobs: None verbose: 0	Cross-validation strategy for generating meta-features

Parameters marked as ”default” refer to the default values of the respective library versions listed above. All experiments were conducted in the same software environment to ensure consistency

## Experimental results

This section covers the results of this study. We started by preprocessing the data, which included the cleaning and normalization, to prepare the data for analysis. Following this, we applied and evaluated 37 ML and DL classifiers, along with the two proposed models. To address data imbalance, we utilized SMOTE oversampling and a hybrid SMOTE-ENN method based on Mahalanobis distance. Finally, we re-evaluated the performance of all 37 classifiers and the two proposed models.

### Performance evaluation of classifiers without data resampling

In this step, we trained 37 machine and deep learning classifiers, along with the two proposed stacking ensemble models, as described in Section 3.3, using the original dataset. All classifiers were implemented with their default settings and parameters. We evaluated their performance using an independent dataset obtained by splitting the original dataset into training and testing sets with a ratio of 80:20. The accuracy, precision, recall, AUC-ROC, and F1-score for all classifiers are summarized in Table  7.

**Table 7:** Evaluation of 37 classifiers and the two proposed models on the original dataset.

Algorithm	Precision	Recall	F1 score	AUC	Accuracy
Naive bayes	0.054	0.816	0.101	0.960	0.978
Decision Tree	0.889	0.459	0.606	0.770	0.999
Logistic Regression	0.829	0.552	0.662	0.970	0.999
Random Forest	0.910	0.701	0.792	0.980	0.999
KNN	0.887	0.724	0.797	0.940	0.999
XGBoost	0.929	0.747	0.828	0.980	0.999
LGBMClassifier	0.329	0.632	0.433	0.750	0.998
LinearDiscriminant Analysis	0.821	0.736	0.776	0.870	0.999
BernoulliNB	0.761	0.586	0.662	0.960	0.999
CatBoost	**0.932**	**0.793**	**0.857**	**0.990**	**0.999**
Neural Network	0.975	0.448	0.614	0.960	0.999
RidgeClassifier	0.725	0.333	0.457	0.980	0.999
AdaBoostClassifier with DecisionTreeClassifier as base	0.761	0.621	0.684	0.990	0.999
AdaBoostClassifier with ExtraTreesClassifier as base	0.943	0.759	0.841	0.960	0.999
AdaBoostClassifier with LogisticRegression as base	0.692	0.414	0.518	0.970	0.999
AdaBoostClassifier with RandomForestClassifier as base	0.940	0.724	0.818	0.950	0.999
AdaBoostClassifier with RidgeClassifier as base	0.727	0.184	0.294	0.960	0.999
ExtraTreesClassifier	0.956	0.747	0.839	0.970	0.999
BaggingClassifier with Decision Trees as base	0.908	0.793	0.847	0.940	0.999
MLP	0.940	0.724	0.818	0.960	0.999
CNN	0.912	0.598	0.722	0.960	0.999
RNN	0.805	0.759	0.781	0.960	0.999
LSTM	0.756	0.747	0.751	0.980	0.999
GRU	0.824	0.701	0.758	0.980	0.999
BiLSTM	0.831	0.736	0.781	0.980	0.999
BiGRU	0.829	0.782	0.805	0.985	0.999
Gradient Boosting	0.862	0.575	0.689	0.790	0.999
Dummy Classifier	0.0	0.0	0.0	0.5	0.999
Quadratic Discriminant Analysis	0.050	0.874	0.095	0.920	0.975
SGDClassifier	0.750	0.448	0.561	0.720	0.999
ANN (Artificial Neural Network)	0.940	0.724	0.818	0.860	0.999
Feedforward Neural Network (FFNN)	0.838	0.713	0.770	0.860	0.999
BiLSTM-Maxpooling	0.857	0.759	0.805	0.988	0.999
BiGRU-Maxpooling	0.952	0.678	0.792	0.987	0.999
Stacking Classifier (RF, CNN, LSTM, XGBoost, Logistic Regression as meta-learner)	0.955	0.724	0.824	0.860	0.999
StackingClassifier (RF, CNN, LSTM, XGBoost as meta-learner)	0.9167	0.759	0.830	0.880	0.999
StackingClassifier (CNN, LSTM, Transformer Base Learners, XGBoost as Meta Learner)	0.753	0.805	0.778	0.90	0.999
The first proposed model: StackingClassifier (ET, CNN, LSTM, XGBoost as meta-learner)	0.955	0.724	0.824	0.86	0.999
The second proposed model: StackingClassifier (ET, AdaBoostClassifier with ExtraTreesClassifier as base, AdaBoostClassifier with RandomForestClassifier as base, XGBoost as meta-learner)	0.878	0.747	0.808	0.87	0.999

As shown in Table  7, the CatBoost classifier achieved the highest accuracy, outperforming all other classifiers. The Accuracy for CatBoost was 0.9996 , Precision was 0.9324, Recall was 0.7931, AUC-ROC was 0.99 while the F1-score was 0.8571.

### Performance evaluation of classifiers after SMOTE resampling and mahalanobis distance SMOTE-ENN hybrid sampling

**Fig. 5:**
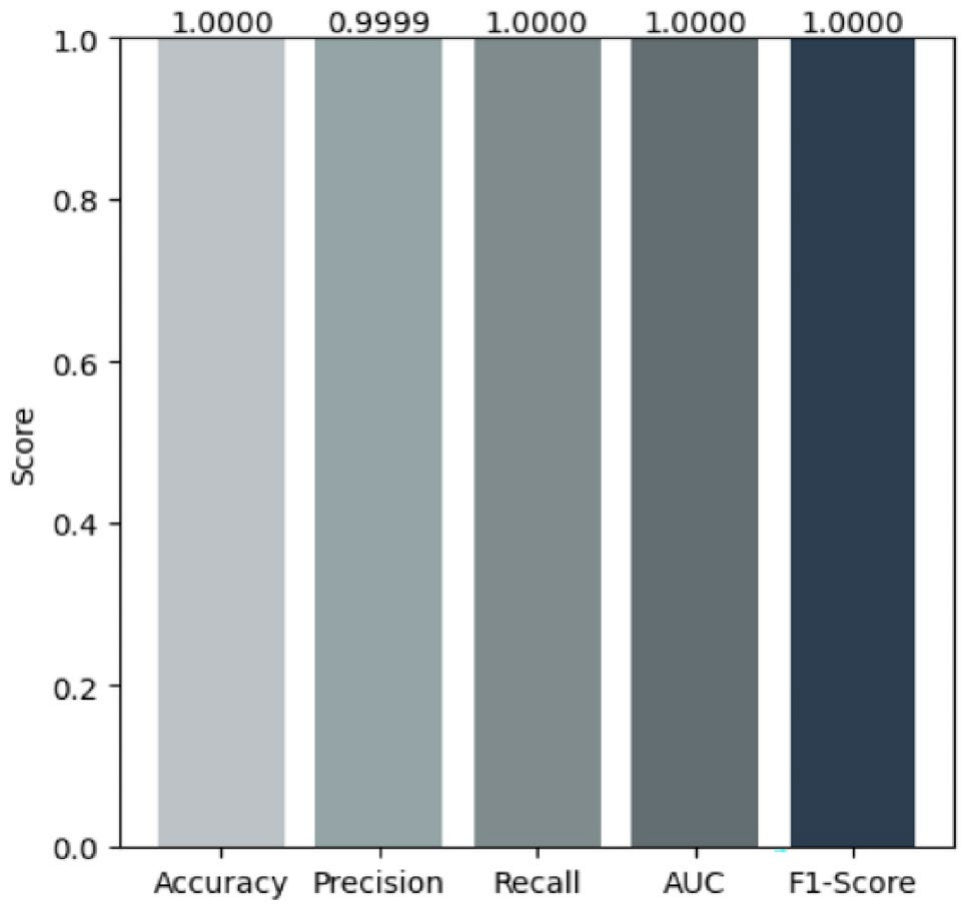
The first proposed stacking ensemble performance metrics using SMOTE.

**Fig. 6:**
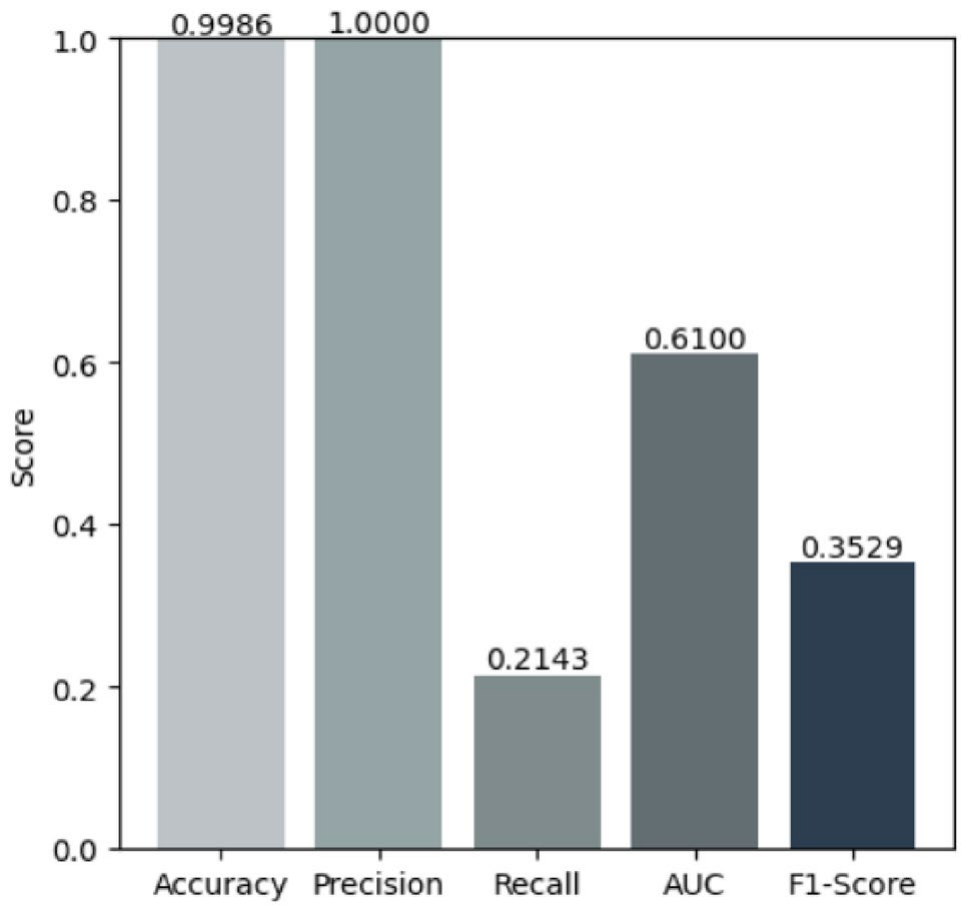
The first proposed stacking ensemble performance metrics using Mahalanobis distance SMOTE-ENN hybrid sampling.

This section presents the experimental results of the classification models listed in Table  3. The results of these algorithms are presented in Table  8. SMOTE oversampling demonstrated superior performance across all evaluation metrics and proved highly effective in managing the imbalanced dataset. Among the individual models tested, CNN achieved an accuracy of 0.9917 with recall of 0.9958, indicating challenges in capturing all fraudulent transactions. LSTM showed improved performance with an accuracy of 0.9983 and recall of 0.9997, demonstrating better capability in capturing temporal dependencies. Extra Trees further enhanced performance with near-perfect results: precision of 0.9998, recall of 1.0, F1 score of 0.9999, and accuracy of 0.9999. XGBoost also achieved strong performance with perfect recall of 1.0 and accuracy of 0.9998, demonstrating its robustness in handling imbalanced datasets. Building upon these strong individual results, we developed two novel stacking ensemble models that achieved superior performance. Tables 5 and 6 provide the parameters for each algorithm used in the proposed stacking ensemble. The first proposed model combines Extra Trees, CNN, and LSTM as base learners with XGBoost as the meta-learner. This ensemble achieved perfect performance with precision of 0.9999, recall of 1.0, F1 score of 1.0, AUC of 1.0, and accuracy of 1.0 (Fig. 5). In contrast, when applying the second balancing method, SMOTE-ENN hybrid sampling with Mahalanobis distance, the model reached an accuracy of 0.9986, precision of 1.0, recall of 0.2143, F1 score of 0.3529, and AUC of 0.6100 (Fig. 6). The second proposed model integrates Extra Trees, AdaBoostClassifier with ExtraTreesClassifier as base, and AdaBoostClassifier with RandomForestClassifier as base, with XGBoost as the meta-learner. This ensemble also achieved perfect performance: precision of 0.9999, recall of 1.0, F1 score of 1.0, AUC of 1.0, and accuracy of 1.0. Both proposed stacking ensemble models demonstrated the best overall results, significantly outperforming individual classifiers. The training and inference procedures of the two proposed stacking ensembles are summarized in Algorithms 1 and 2.

**Table 8:** Evaluation of 37 classifiers and the two proposed models on the balanced dataset.

Algorithm	Imbalance method	Precision	Recall	F1 score	AUC	Accuracy
Naive Bayes	SMOTE	0.969	0.848	0.905	0.950	0.910
	Mahalanobis distance SMOTE-ENN hybrid sampling	0.052	0.878	0.098	0.960	0.972
Decision Tree	SMOTE	0.994	0.878	0.933	0.960	0.936
	Mahalanobis distance SMOTE-ENN hybrid sampling	0.079	0.806	0.145	0.880	0.984
Logistic Regression	SMOTE	0.974	0.919	0.946	0.990	0.947
	Mahalanobis distance SMOTE-ENN hybrid sampling	0.059	0.918	0.111	0.970	0.975
Random Forest	SMOTE	0.995	0.925	0.959	0.990	0.960
	Mahalanobis distance SMOTE-ENN hybrid sampling	0.899	0.816	0.856	0.970	0.999
KNN	SMOTE	0.997	1.0	0.999	1.0	0.999
	Mahalanobis distance SMOTE-ENN hybrid sampling	0.566	0.878	0.688	0.940	0.999
XGBoost	SMOTE	0.999	1.0	0.999	1.0	0.999
	Mahalanobis distance SMOTE-ENN hybrid sampling	0.837	0.837	0.837	0.970	0.999
LGBM Classifier	SMOTE	0.999	0.999	0.999	1.0	0.999
	Mahalanobis distance SMOTE-ENN hybrid sampling	0.778	0.857	0.816	0.950	0.999
Linear Discriminant Analysis	SMOTE	0.983	0.849	0.911	0.980	0.917
	Mahalanobis distance SMOTE-ENN hybrid sampling	0.094	0.847	0.169	0.960	0.986
BernoulliNB	SMOTE	0.989	0.822	0.898	0.960	0.906
	Mahalanobis distance SMOTE-ENN hybrid sampling	0.159	0.837	0.267	0.960	0.992
CatBoost	SMOTE	0.999	1.0	0.999	1.0	0.999
	Mahalanobis distance SMOTE-ENN hybrid sampling	0.746	0.867	0.802	0.970	0.999
Neural Network	SMOTE	0.999	1.0	0.999	1.0	0.999
	Mahalanobis distance SMOTE-ENN hybrid sampling	0.649	0.867	0.742	0.960	0.999
Ridge Classifier	SMOTE	0.983	0.849	0.911	0.980	0.917
	Mahalanobis distance SMOTE-ENN hybrid sampling	0.094	0.847	0.169	0.960	0.986
AdaBoost Classifier with DecisionTree Classifier as base	SMOTE	0.976	0.950	0.963	1.0	0.963
	Mahalanobis distance SMOTE-ENN hybrid sampling	0.083	0.898	0.152	0.970	0.983
AdaBoost Classifier with ExtraTrees Classifier as base	SMOTE	0.999	1.0	0.999	1.0	0.999
	Mahalanobis distance SMOTE-ENN hybrid sampling	0.921	0.827	0.871	0.960	0.999
AdaBoost Classifier with Logistic Regression as base	SMOTE	0.974	0.899	0.935	0.980	0.937
	Mahalanobis distance SMOTE-ENN hybrid sampling	0.049	0.888	0.093	0.970	0.970
AdaBoost Classifier with RandomForest Classifier as base	SMOTE	0.999	1.0	0.999	1.0	0.999
	Mahalanobis distance SMOTE-ENN hybrid sampling	0.907	0.796	0.848	0.950	0.999
AdaBoost Classifier with Ridge Classifier as base	SMOTE	0.986	0.879	0.930	0.980	0.934
	Mahalanobis distance SMOTE-ENN hybrid sampling	0.081	0.888	0.149	0.960	0.985
ExtraTrees Classifier	SMOTE	0.999	1.0	0.999	1.0	0.999
	Mahalanobis distance SMOTE-ENN hybrid sampling	0.921	0.827	0.871	0.960	0.999
Bagging Classifier with Decision Trees as base	SMOTE	0.999	0.999	0.999	1.0	0.999
	Mahalanobis distance SMOTE-ENN hybrid sampling	0.784	0.775	0.779	0.910	0.999
MLP	SMOTE	0.999	1.0	0.999	1.0	0.999
	Mahalanobis distance SMOTE-ENN hybrid sampling	0.689	0.837	0.756	0.960	0.999
CNN	SMOTE	0.988	0.996	0.992	0.999	0.992
	Mahalanobis distance SMOTE-ENN hybrid sampling	0.169	0.898	0.284	0.969	0.992
RNN	SMOTE	0.994	0.998	0.996	0.999	0.996
	Mahalanobis distance SMOTE-ENN hybrid sampling	0.215	0.867	0.344	0.940	0.994
LSTM	SMOTE	0.997	0.999	0.998	0.999	0.998
	Mahalanobis distance SMOTE-ENN hybrid sampling	0.449	0.857	0.589	0.965	0.998
GRU	SMOTE	0.996	0.999	0.998	0.999	0.998
	Mahalanobis distance SMOTE-ENN hybrid sampling	0.483	0.867	0.620	0.952	0.998
BiLSTM	SMOTE	0.998	0.999	0.999	0.999	0.999
	Mahalanobis distance SMOTE-ENN hybrid sampling	0.607	0.867	0.714	0.955	0.998
BiGRU	SMOTE	0.999	0.999	0.999	0.999	0.999
	Mahalanobis distance SMOTE-ENN hybrid sampling	0.659	0.867	0.749	0.958	0.999
Gradient Boosting	SMOTE	0.987	0.967	0.977	0.977	0.977
	Mahalanobis distance SMOTE-ENN hybrid sampling	0.176	0.898	0.294	0.945	0.993
Dummy Classifier	SMOTE	0.0	0.0	0.0	0.5	0.498
	Mahalanobis distance SMOTE-ENN hybrid sampling	0.002	1.0	0.003	0.5	0.002
Quadratic Discriminant Analysis	SMOTE	0.966	0.886	0.924	0.927	0.927
	Mahalanobis distance SMOTE-ENN hybrid sampling	0.045	0.888	0.085	0.927	0.967
SGDClassifier	SMOTE	0.972	0.922	0.947	0.947	0.947
	Mahalanobis distance SMOTE-ENN hybrid sampling	0.061	0.918	0.115	0.947	0.976
ANN (Artificial Neural Network)	SMOTE	0.999	1.0	0.999	0.999	0.999
	Mahalanobis distance SMOTE-ENN hybrid sampling	0.717	0.827	0.767	0.913	0.999
Feedforward Neural Network (FFNN)	SMOTE	0.999	1.0	0.999	0.999	0.999
	Mahalanobis distance SMOTE-ENN hybrid sampling	0.728	0.847	0.783	0.923	0.999
BiLSTM-Maxpooling	SMOTE	0.999	1.0	0.999	0.999	0.999
	Mahalanobis distance SMOTE-ENN hybrid sampling	0.593	0.847	0.698	0.973	0.999
BiGRU-Maxpooling	SMOTE	0.999	0.998	0.999	0.999	0.999
	Mahalanobis distance SMOTE-ENN hybrid sampling	0.737	0.857	0.793	0.955	0.9992
Stacking Classifier (RF, CNN, LSTM, XGBoost, Logistic Regression as meta-learner)	SMOTE	0.999	1.0	0.999	0.999	0.999
	Mahalanobis distance SMOTE-ENN hybrid sampling	0.847	0.847	0.847	0.920	0.999
Stacking Classifier (RF, CNN, LSTM, XGBoost as meta-learner)	SMOTE	0.999	1.0	0.999	0.999	0.999
	Mahalanobis distance SMOTE-ENN hybrid sampling	0.944	0.347	0.508	0.670	0.999
Stacking Classifier (CNN, LSTM, Transformer Base Learners, XGBoost as Meta Learner)	SMOTE	0.999	0.994	0.997	0.997	0.997
	Mahalanobis distance SMOTE-ENN hybrid sampling	0.789	0.837	0.812	0.920	0.999
The first proposed model	SMOTE	**0.999**	**1.0**	**1.0**	**1.0**	**1.0**
	Mahalanobis distance SMOTE-ENN hybrid sampling	1.0	0.214	0.353	0.610	0.999
The second proposed model	SMOTE	**0.999**	**1.0**	**1.0**	**1.0**	**1.0**
	Mahalanobis distance SMOTE-ENN hybrid sampling	1.0	0.225	0.367	0.612	0.999

**Algorithm 1:**
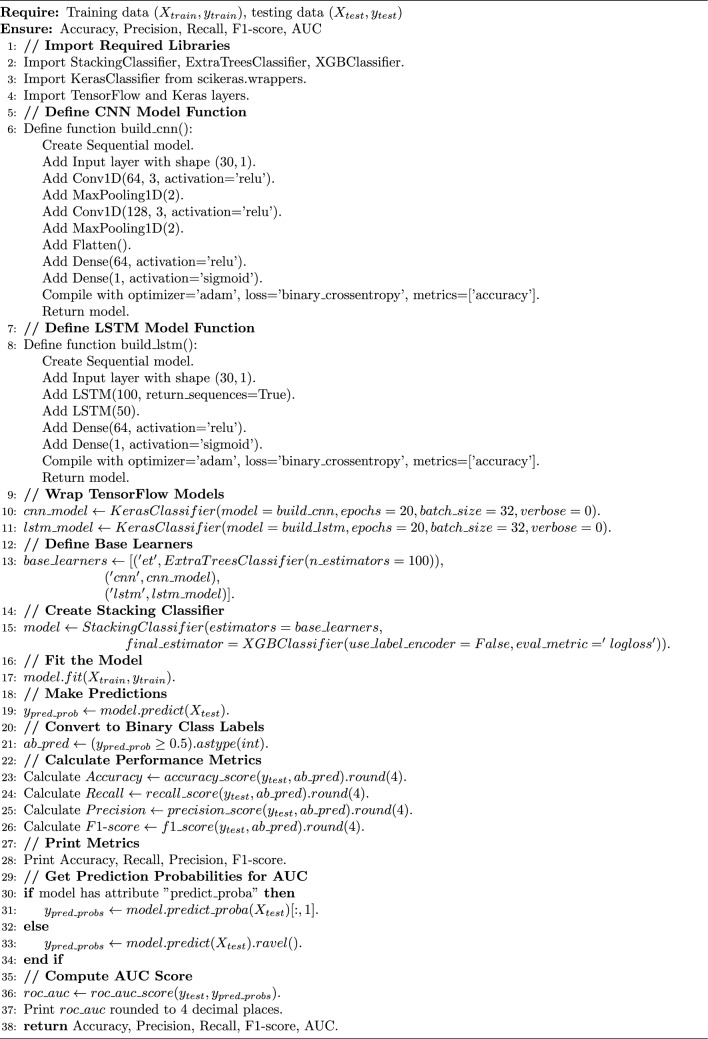
First proposed model: Extra Trees–CNN–LSTM stacking ensemble with XGBoost

**Algorithm 2:**
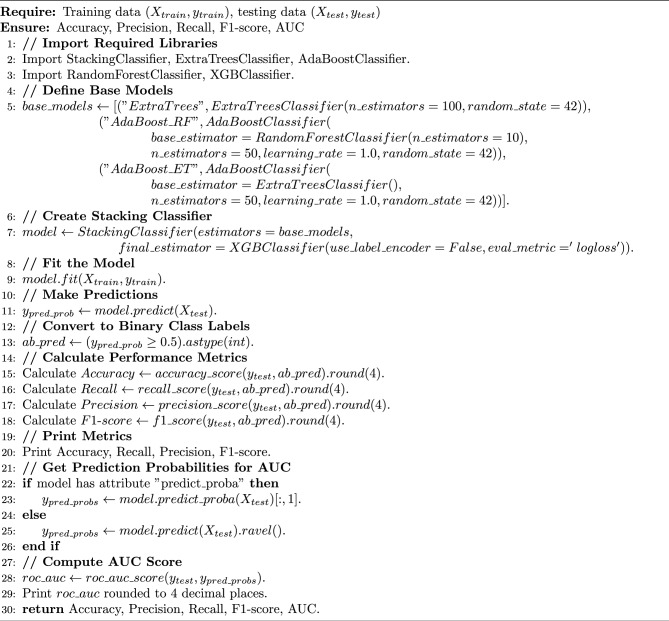
Second proposed model: ensemble boosting stacking with XGBoost meta-learner

### Discussion of simpler baselines

Although our primary focus was on ensemble methods, we also included several simpler baseline models such as Logistic Regression, Decision Tree, k-NN, and Naïve Bayes in our evaluation. These models are widely recognized for their efficiency and interpretability, but as our results demonstrate, they consistently underperform compared to the proposed ensembles, particularly in detecting fraudulent transactions.

This highlights an important trade-off: simpler models are computationally lighter and easier to explain, whereas ensemble approaches achieve substantially higher accuracy and robustness. While we did not explicitly measure the computational efficiency of the simpler baselines in this study, their advantages in this regard are well established in the literature. A detailed comparative analysis of efficiency across both simple and complex models is left as a promising direction for future work.

### Comparison with other studies

The performance of the proposed model was compared to the most effective methods from previous studies, as shown in Table  8. The results indicate that our model outperforms the methods presented in other studies. Specifically, by comparing these metrics, it is clear that the combination of SMOTE oversampling and the proposed stacking ensemble (which incorporates ExtraTrees, CNN, LSTM, and XGBoost as a meta-learner or incorporates ExtraTrees, AdaBoostClassifier with ExtraTreesClassifier as base, AdaBoostClassifier with RandomForestClassifier as base and XGBoost as meta-learner) is more effective in predicting fraud risk.

**Table 9:** Comparison with studies that used european credit card dataset.

Refs.	Pre-processing	Method	Results without data resampling	Results with data resampling
^10^	SMOTE-ENN	Proposed DL En-semble	sensitivity= 0.905 specificity = 0.966	sensitivity= 1.000 specificity = 0.997
^11^	NA	Decision Tree	Sensitivity= 0.792 Precision= 0.851	NA
^36^	SMOTE, GAN, VAE, VAEGAN and The improved VAEGAN	XGBoost with The improved VAEGAN	Precision =0.958 Recall =0.704 F1_score =0.812 Specificity =0.999	Precision =0.924 Recall =0.839 F1_score =0.879 Specificity =0.999
^46^	feature selection techniques, removing non fraudulent transactions from the dataset.	the proposed 20-layer CNN model	Accuracy =0.932	Accuracy =0.997
^16^	SMOTE-ENN	Proposed LSTM Ensemble	Sensitivity = 0.839 Specificity =0.982 AUC =0.890	Sensitivity =0.996 Specificity =0.998 AUC =0.990
^7^	PCA And EDA	Random Forest and XGBoost	Not mentioned	RF Accuracy =0.986 XGBoost Accuracy =0.984
^8^	OneHotEncoding, PCA, Silhouette Scoring, IFM, SMOTE	RXT-J (proposed)	Not mentioned	Accuracy =0.981
^3^	feature selection, class weight hyperparameter tuning to deal with the problem of data unbalance	Proposed LightGBM , Proposed approach	Not mentioned	Proposed LightGBM Accuracy = 0.999 Proposed approach Accuracy = 0.999
^12^	GANs	GANs	Not mentioned	AUC = 0.989
^1^	PCA , random under-sampling	LR	Not mentioned	Accuracy =0.959
^9^	Feature Scaling, undersampling with borderline SMOTE	SVM with undersampling with borderline SMOTE	Accuracy =0.860	Accuracy =0.938
^47^	SMOTE	XGB-AdaBoost and ET-AdaBoost	Not mentioned	Accuracy of XGB-AdaBoost ET-AdaBoost = 99.98
Ours	SMOTE	Stacking Classifier (ET, CNN, LSTM, XGBoost as meta-learner)	Precision = 0.955 Recall= 0.724 F1 score = 0.824 AUC= 0.860 Accuracy= 0.999	Precision = **0.9999** Recall= **1.0** F1 score= **1.0** AUC= **1.0** Accuracy= **1.0**
		Stacking Classifier (ET, AdaBoostClassifier with ExtraTreesClassifier as base, AdaBoostClassifier with RandomForestClassifier as base, XGBoost as meta-learner)	Precision = 0.878 Recall= 0.747 F1 score = 0.808 AUC= 0.870 Accuracy= 0.999	Precision =** 0.9999** Recall= **1.0** F1 score= **1.0** AUC= **1.0** Accuracy= **1.0**

## Computational cost analysis

This section evaluates the computational efficiency of the two proposed ensemble models for credit card fraud detection. The computational cost analysis includes training time and peak memory usage across different dataset sizes.

### Computational cost comparison

**Fig. 7:**
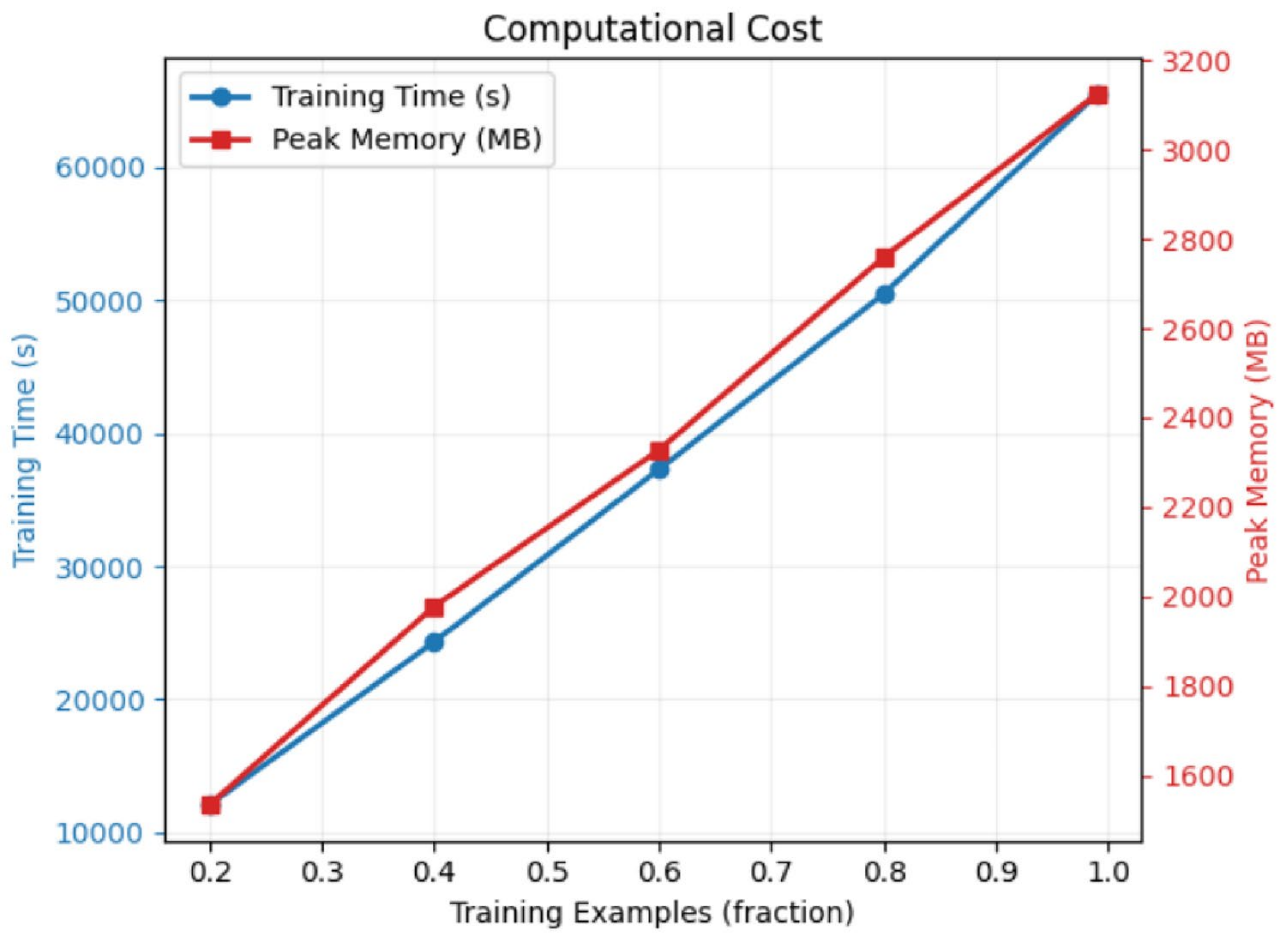
Computational cost for Model 1 (Deep Ensemble Stack).

**Fig. 8:**
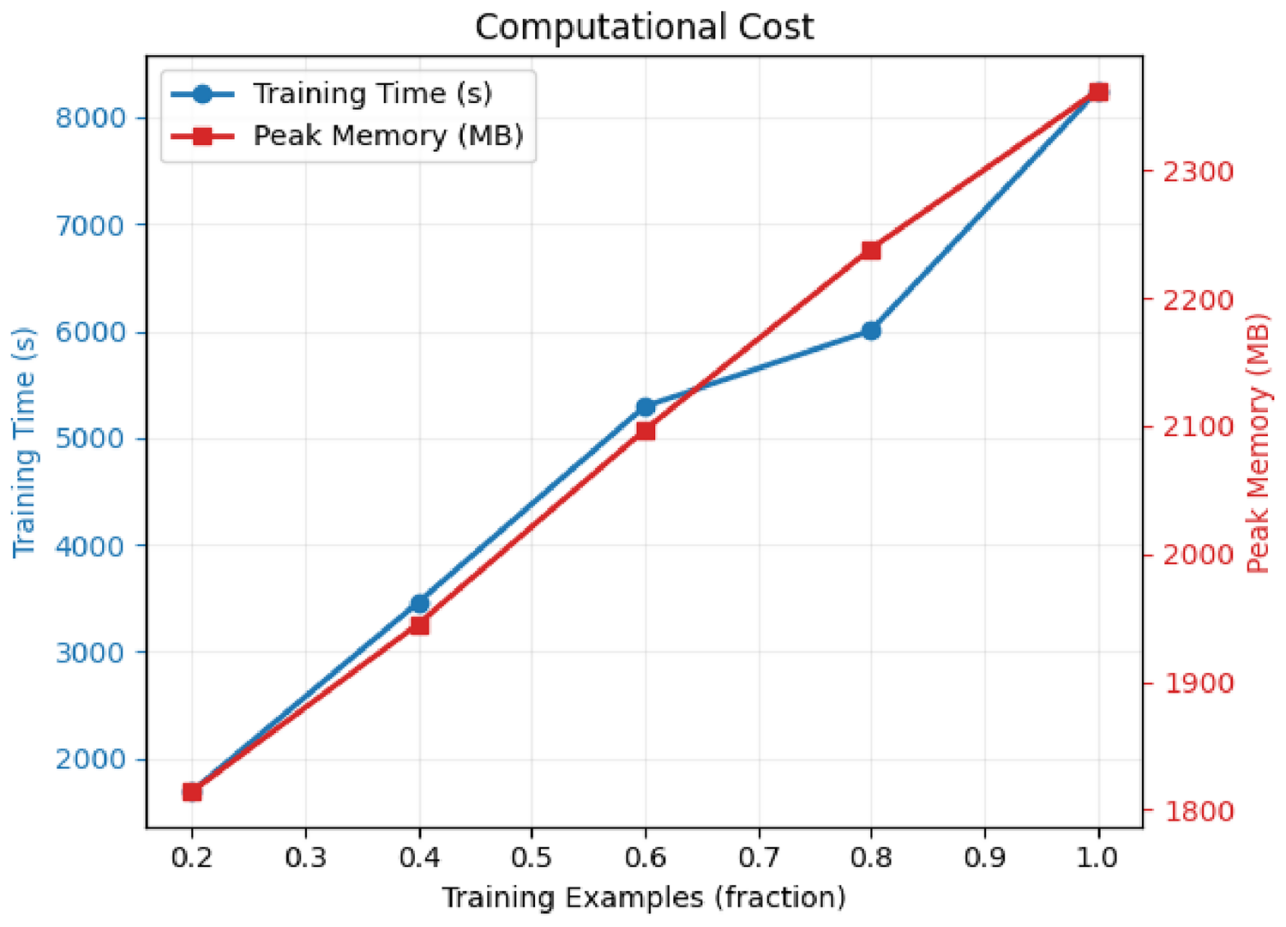
Computational cost for Model 2 (Tree Ensemble).

Figures 7 and  8 illustrate the computational requirements of both proposed models across different dataset sizes, while Table 9 provides detailed numerical comparisons.

**Table 10:** Computational cost comparison between proposed models.

Dataset fraction	Model 1 (deep ensemble)		Model 2 (tree ensemble)	
	Time (s)	Memory (MB)	Time (s)	Memory (MB)
0.2	12,000	1600	1800	1800
0.4	25,000	2000	3500	1950
0.6	38,000	2350	5200	2100
0.8	51,000	2800	6000	2250
1.0	65,000	3200	8200	2350

### Computational efficiency analysis

#### Training time complexity

The computational cost analysis reveals significant differences between the two proposed models:

- Model 1 (Deep Ensemble): Exhibits substantial computational overhead due to deep neural network components. Training time scales from 12,000s (0.2 fraction) to 65,000s (full dataset), representing a 5.4$\times$ increase.
- Model 2 (Tree Ensemble): Demonstrates superior computational efficiency with training times ranging from 1,800s to 8,200s, achieving a 4.6$\times$ increase with dataset size.
- Efficiency Ratio: Model 2 achieves approximately 87% faster training time compared to Model 1 across all dataset sizes, with the efficiency gap remaining consistent.

#### Memory utilization

Memory consumption patterns differ significantly between the models:

- Model 1: Peak memory usage ranges from 1,600MB to 3,200MB, showing a 2$\times$ increase with full dataset utilization. The neural network components require substantial memory for gradient computations and model parameters.
- Model 2: More memory-efficient with usage ranging from 1,800MB to 2,350MB, representing only a 1.3$\times$ increase. Tree-based algorithms demonstrate better memory scalability.
- Memory Overhead: Model 1 requires 36% more peak memory at full dataset capacity, primarily due to CNN and LSTM layer computations.

#### Scalability assessment

Both models exhibit near-linear scaling characteristics, but with different computational complexity coefficients:

7 $$\begin{aligned} T_{Model1} \approx 65,000 \times f_{data} + C_1 \end{aligned}$$

8 $$\begin{aligned} T_{Model2} \approx 8,200 \times f_{data} + C_2 \end{aligned}$$

where $f_{data}$ represents the data fraction and $C_1$, $C_2$ are model-specific constants.

### Practical deployment implications

#### Real-time processing considerations

For production deployment in credit card fraud detection systems:

- Model 2 offers superior suitability for environments with computational constraints or real-time processing requirements
- Model 1 may be preferable when computational resources are abundant and maximum predictive performance is prioritized over efficiency
- Both models can be deployed in distributed computing environments, but Model 2 requires significantly fewer computational resources

#### Cost-benefit analysis

The computational efficiency of Model 2 provides several operational advantages:

- Reduced infrastructure costs due to lower computational requirements
- Faster model retraining capabilities for adapting to new fraud patterns
- Lower energy consumption and carbon footprint
- Enhanced scalability for processing large transaction volumes

## Model generalization assessment for proposed ensemble models

To address concerns about potential overfitting given the exceptionally high performance metrics, we conducted comprehensive learning curve analyses for both of our proposed ensemble models. Learning curves provide crucial evidence of model generalization by visualizing training and validation performance across different training set sizes.

### Learning curve analysis

**Fig. 9:**
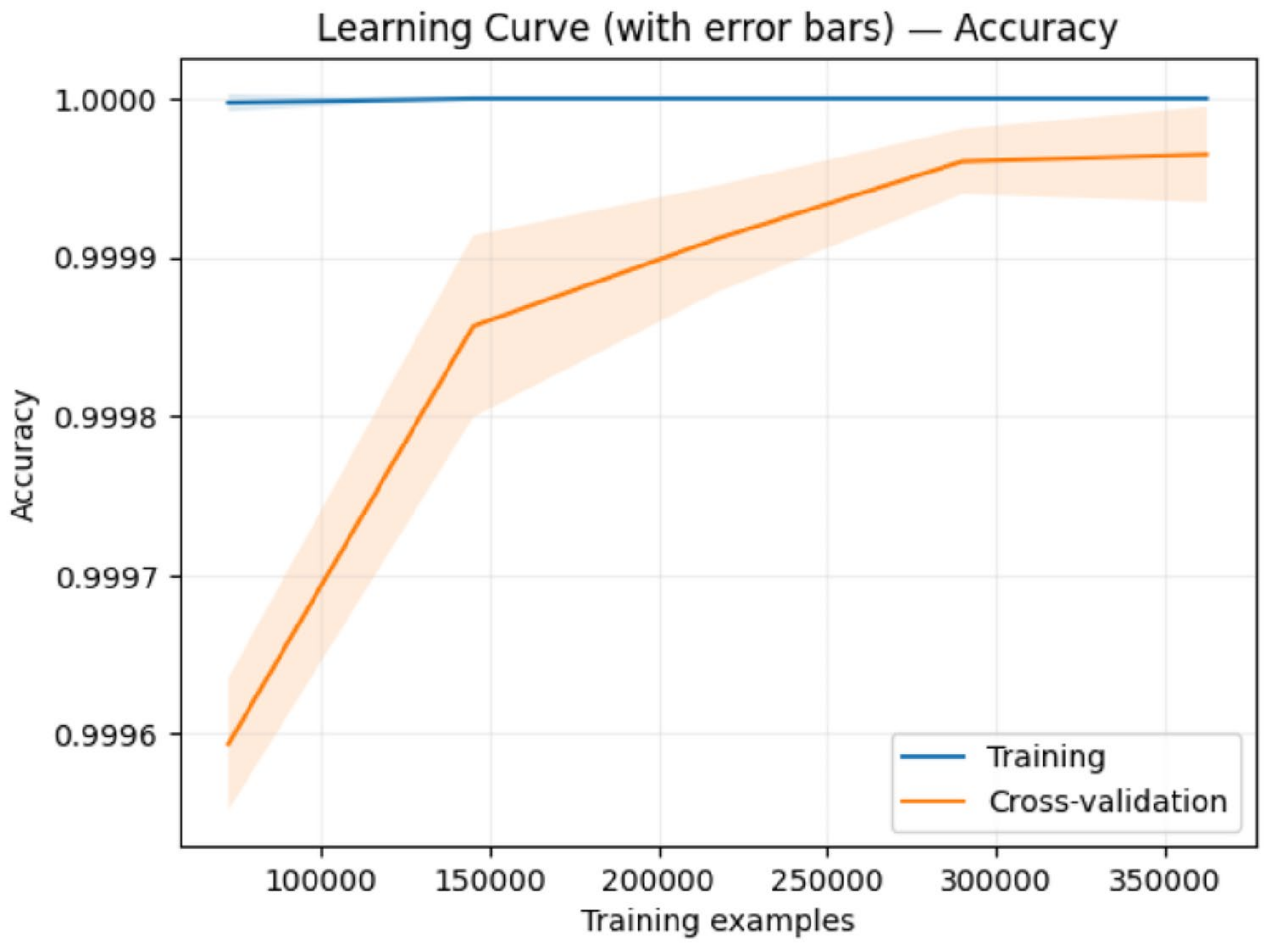
Learning curve with error bars for the first proposed ensemble model showing minimal training-validation gap and decreasing variance with larger training sets.

**Fig. 10:**
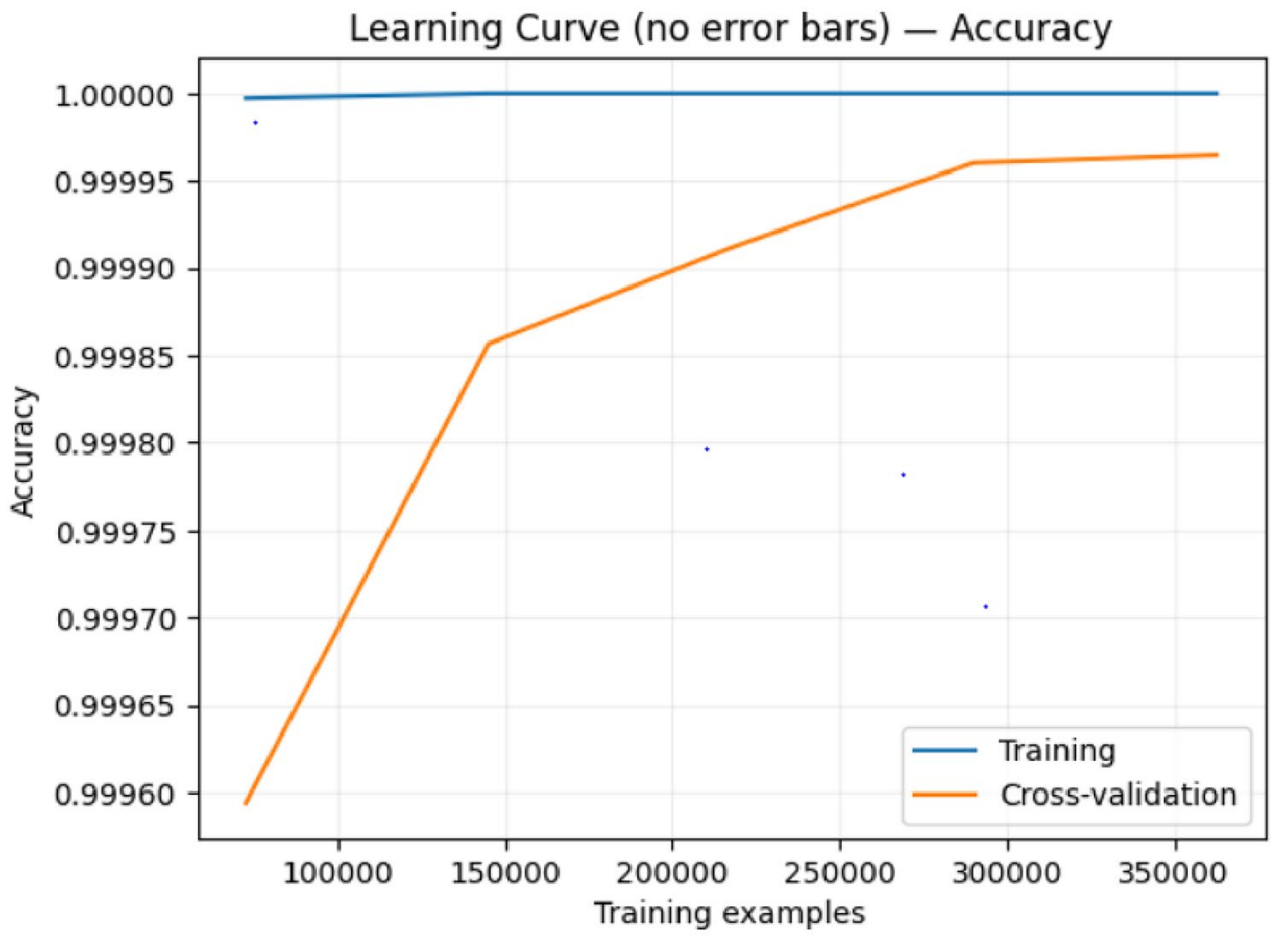
Learning curve for the first proposed model demonstrating consistent validation improvement without overfitting.

**Fig. 11:**
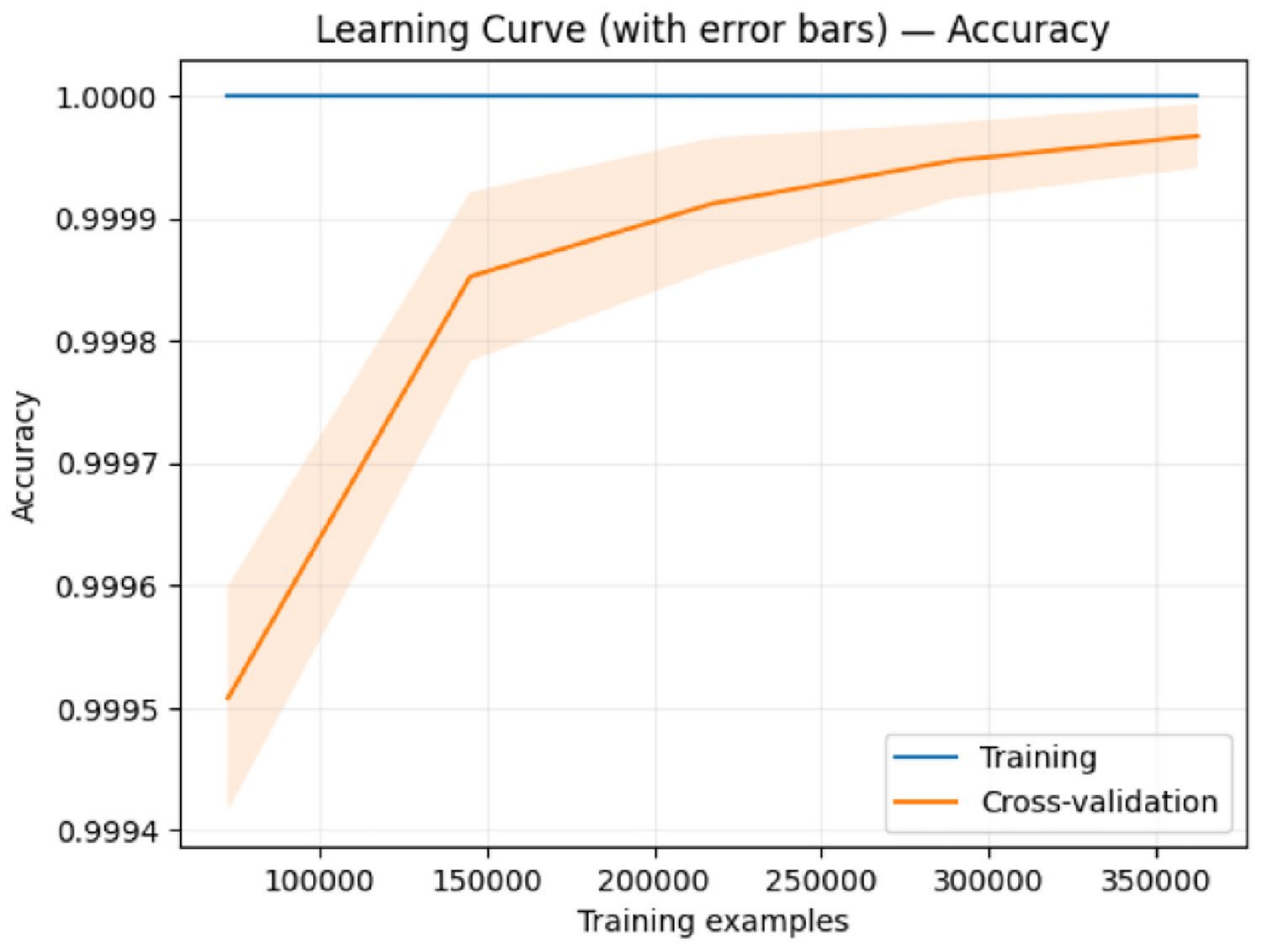
Learning curve with error bars for the second proposed ensemble model showing rapid early convergence and stable generalization.

**Fig. 12:**
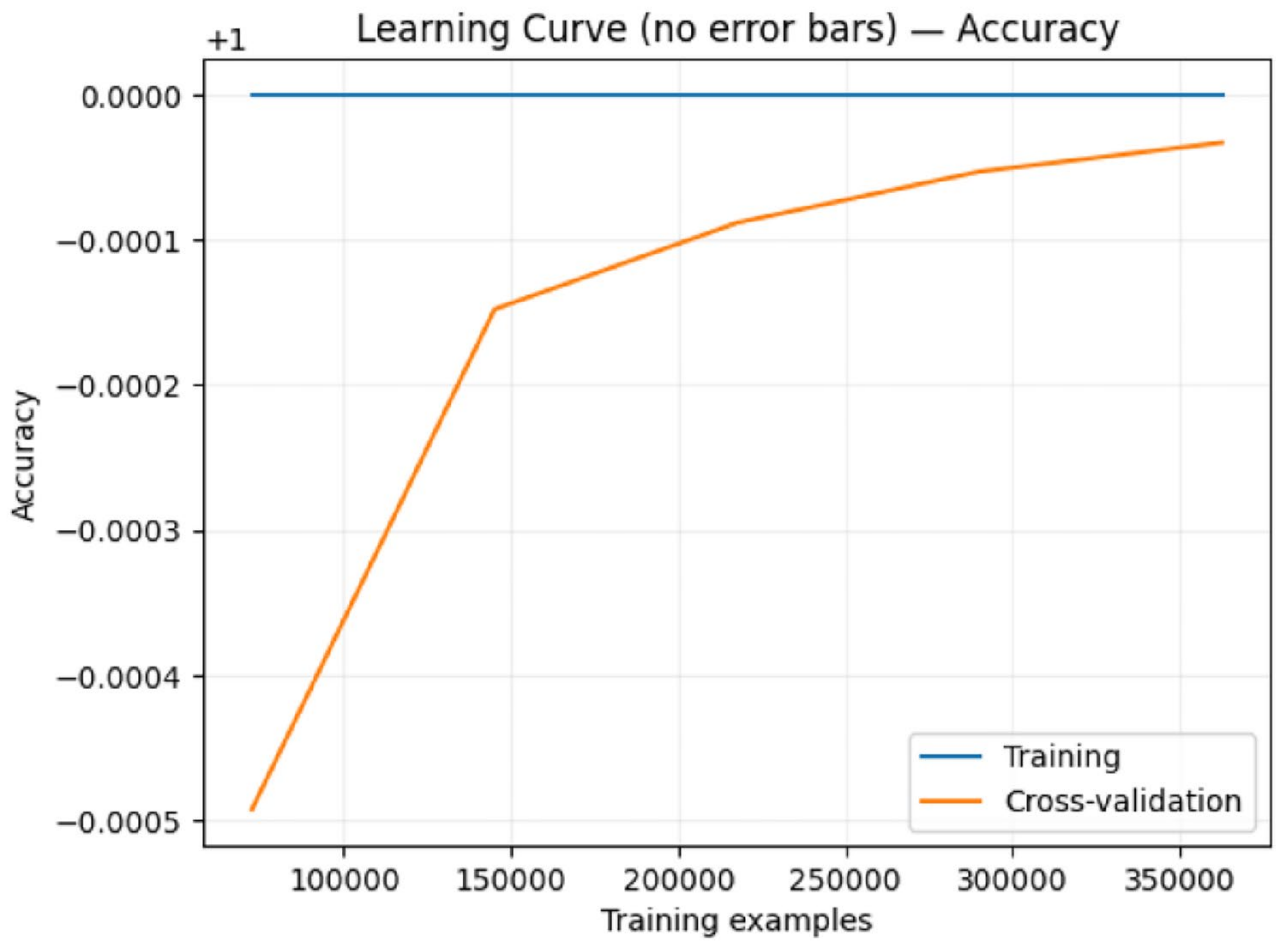
Learning curve for the second proposed model demonstrating efficient learning with fast convergence to optimal performance.

Figures 9, 10, 11, and 12 present the learning curves for our first and second proposed ensemble models, respectively. Both analyses examine accuracy performance across training set sizes ranging from 80,000 to 350,000 samples. Model 1 Performance: Training accuracy remains consistently at 1.0000 while cross-validation accuracy improves from 0.9995 to 0.9999 as training data increases. The final gap between training and validation curves is only 0.0001 (0.01%). The error bars in Figure 9 show small standard deviations that decrease with larger training sets, indicating stable performance estimates.

Model 2 Performance: Training accuracy maintains perfect 1.0000, while cross-validation accuracy shows a steeper initial improvement, rising from 0.9995 to 0.9999. The model demonstrates more rapid convergence around 150,000 training samples, with a final gap of approximately 0.0001, matching Model 1’s convergence behavior.

### Comparative analysis

The two models show distinct learning patterns:

- Convergence Speed: Model 2 achieves faster early convergence (80,000–150,000 samples), while Model 1 shows gradual, consistent improvement throughout.
- Data Efficiency: Model 2 reaches near-optimal performance with smaller training sets, making it suitable for limited data scenarios.
- Final Performance: Both models achieve identical convergence gaps (0.0001), indicating comparable generalization capabilities.
- Stability: Both demonstrate smooth learning curves with minimal variance across cross-validation folds.

### Overfitting assessment

The learning curves provide strong evidence against overfitting:

The minimal training-validation gaps ($\le$ 0.01%) are far below typical overfitting indicators. Both models show progressive validation improvement rather than the characteristic overfitting pattern where validation performance plateaus while training performance continues improving. The decreasing error bars with larger training sets indicate increasing confidence in performance estimates, and the healthy convergence patterns demonstrate genuine learning rather than memorization.

### Implications for fraud detection applications

The results have practical implications for deployment:

1. Production Reliability: Minimal overfitting ensures both models will maintain performance on new fraud cases.
2. Resource Optimization: Model 2’s faster convergence suits scenarios with limited training data, while Model 1’s consistent improvement benefits from abundant data.
3. Deployment Confidence: The 0.01% performance gaps validate that reported metrics (>99.9% accuracy) reflect genuine capabilities rather than artifacts.

These findings establish that our exceptionally high performance metrics represent legitimate model capabilities with strong generalization to real-world fraud detection scenarios.

## Explainable AI (XAI) analysis

This section provides a comprehensive analysis of the explainability results for two proposed credit card fraud detection models using SHAP (SHapley Additive exPlanations) and LIME (Local Interpretable Model-agnostic Explanations) techniques. The analysis demonstrates the interpretability and transparency of both models through various visualization approaches.

### Model 1: Explainability analysis

The first proposed model’s explainability is analyzed through multiple XAI techniques, providing insights into its decision-making process and feature importance patterns.

#### LIME analysis

**Fig. 13:**
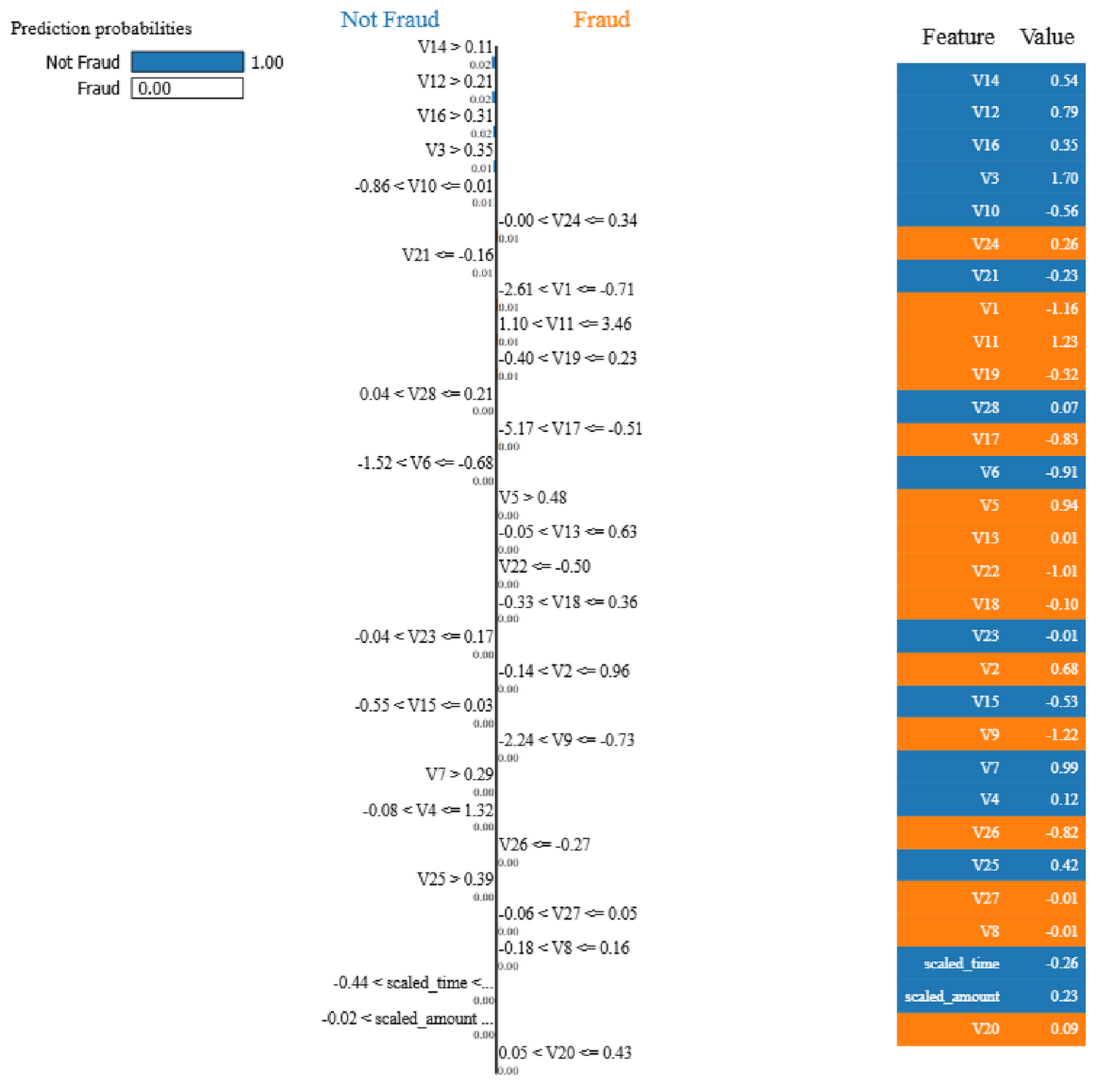
LIME visualization for Model 1 showing prediction probabilities and feature thresholds.

The LIME analysis reveals the hierarchical decision-making process of Model 1, as illustrated in Figure 13. The model achieves perfect classification confidence with prediction probabilities of 1.00 for Not Fraud and 0.00 for Fraud for the analyzed instance. The decision tree structure demonstrates clear logical progression through key features:

The primary decision nodes include splits on features V14 (> 0.11), V12 (> 0.21), V16 (> 0.31), and V3 (> 0.35), with specific threshold values that guide the classification process. The feature value panel shows the actual values for each feature in the analyzed instance, providing transparency in the decision-making process.

#### SHAP analysis

**Fig. 14:**
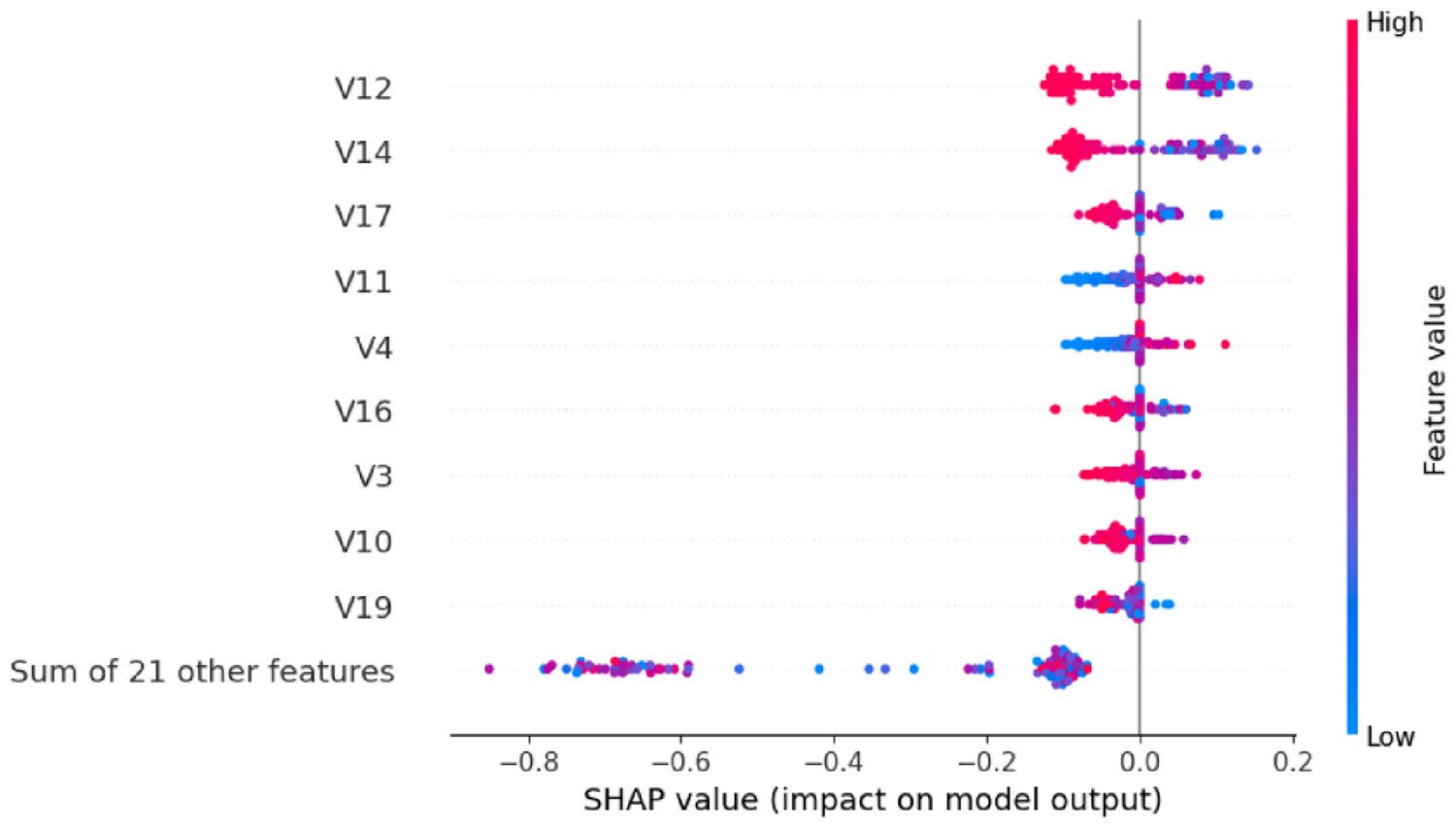
SHAP beeswarm plot for Model 1 showing feature importance and directional impact on predictions.

The SHAP analysis for Model 1 provides multiple perspectives on feature importance and contribution patterns. Figure 14 presents the beeswarm plot showing the relationship between feature values and their impact on model predictions.

The beeswarm visualization reveals that V12, V14, V17, V11, V4, V16, V3, V10, and V19 are the most influential features. The color coding indicates feature values (red for high, blue for low), while the x-axis position shows the magnitude and direction of impact on the model output.

**Fig. 15:**
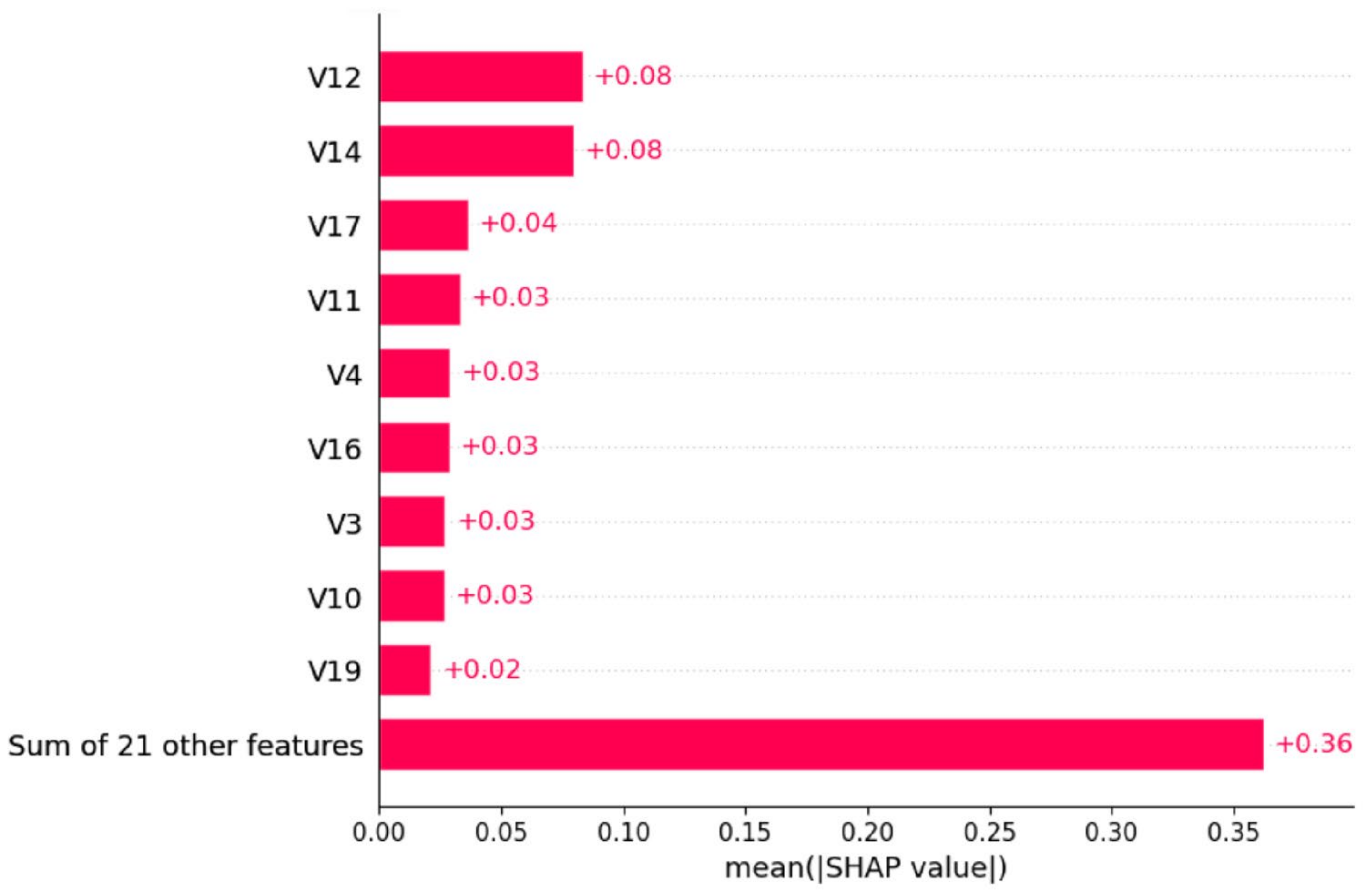
SHAP feature importance ranking for Model 1 with mean absolute SHAP values.

Figure 15 quantifies the feature importance through mean absolute SHAP values:

The analysis reveals that V12 and V14 demonstrate the highest mean SHAP values (0.08 each), indicating their strongest predictive power. Secondary features including V17, V11, V4, V16, V3, V10, and V19 contribute moderately with values ranging from 0.02 to 0.04. Notably, the sum of 21 other features collectively contributes 0.36, suggesting the model leverages a comprehensive feature space.

**Fig. 16:**
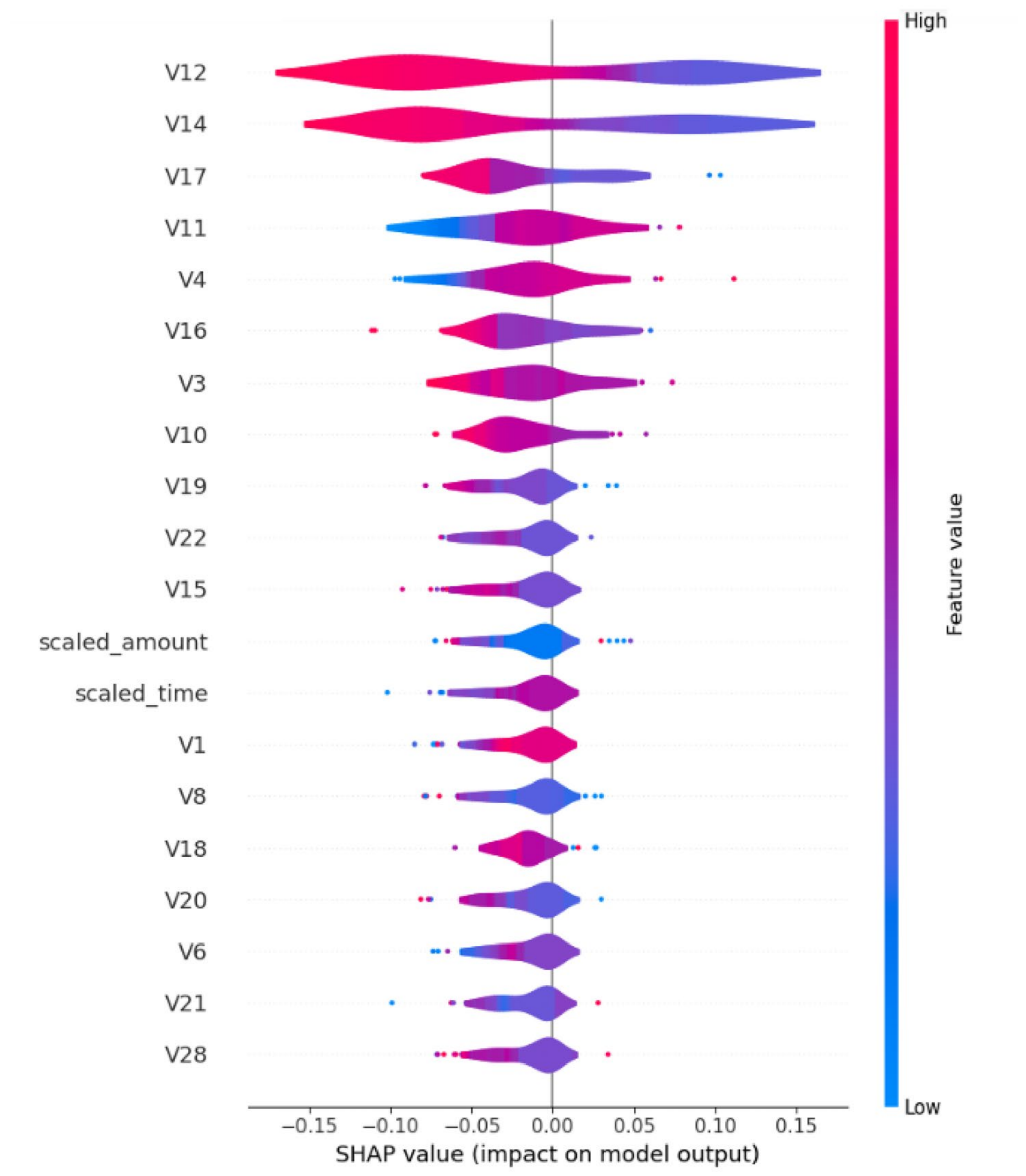
SHAP violin plot for Model 1 showing the distribution of feature impact values.

The distribution of feature impacts is further illustrated in Fig. 16:

The violin plot demonstrates the variability in feature contributions across different instances, with V12, V14, V17, V11, and V4 showing the most substantial and consistent impacts on model predictions.

### Model 2: Explainability analysis

The second proposed model exhibits enhanced complexity and refined feature utilization patterns, as demonstrated through comprehensive XAI analysis.

#### LIME analysis

**Fig. 17:**
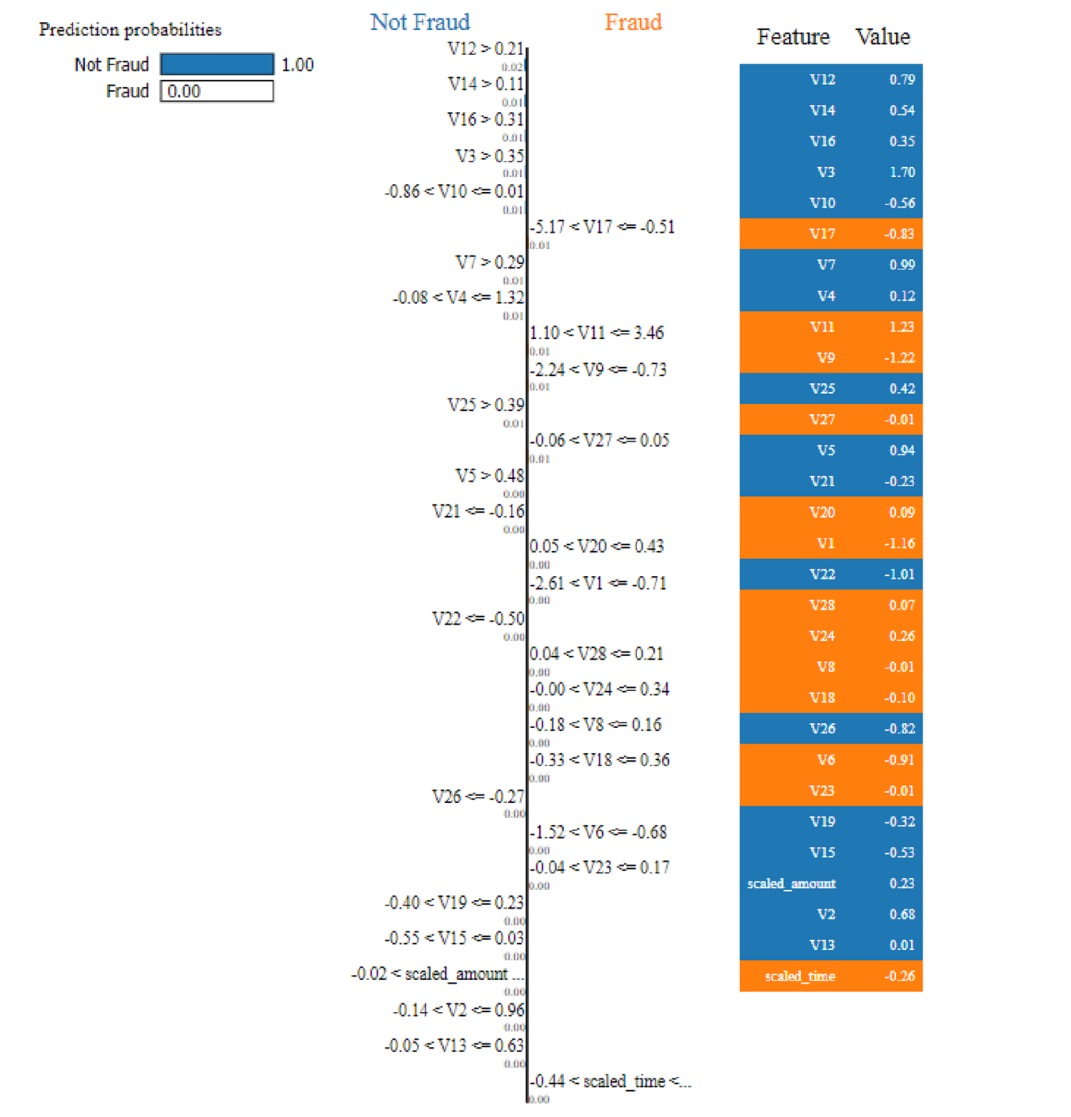
LIME visualization for Model 2 showing enhanced decision complexity.

The LIME analysis for Model 2, shown in Fig. 17, reveals a more sophisticated decision tree structure with additional branching levels This enhanced LIME surrogate structure suggests that the underlying complex model requires deeper feature interactions for local explanations and more nuanced decision boundaries. The LIME explanation maintains high confidence levels with similar prediction probabilities (1.00 for Not Fraud) while incorporating multiple pathways for local classification through various feature combinations.

#### SHAP analysis

**Fig. 18:**
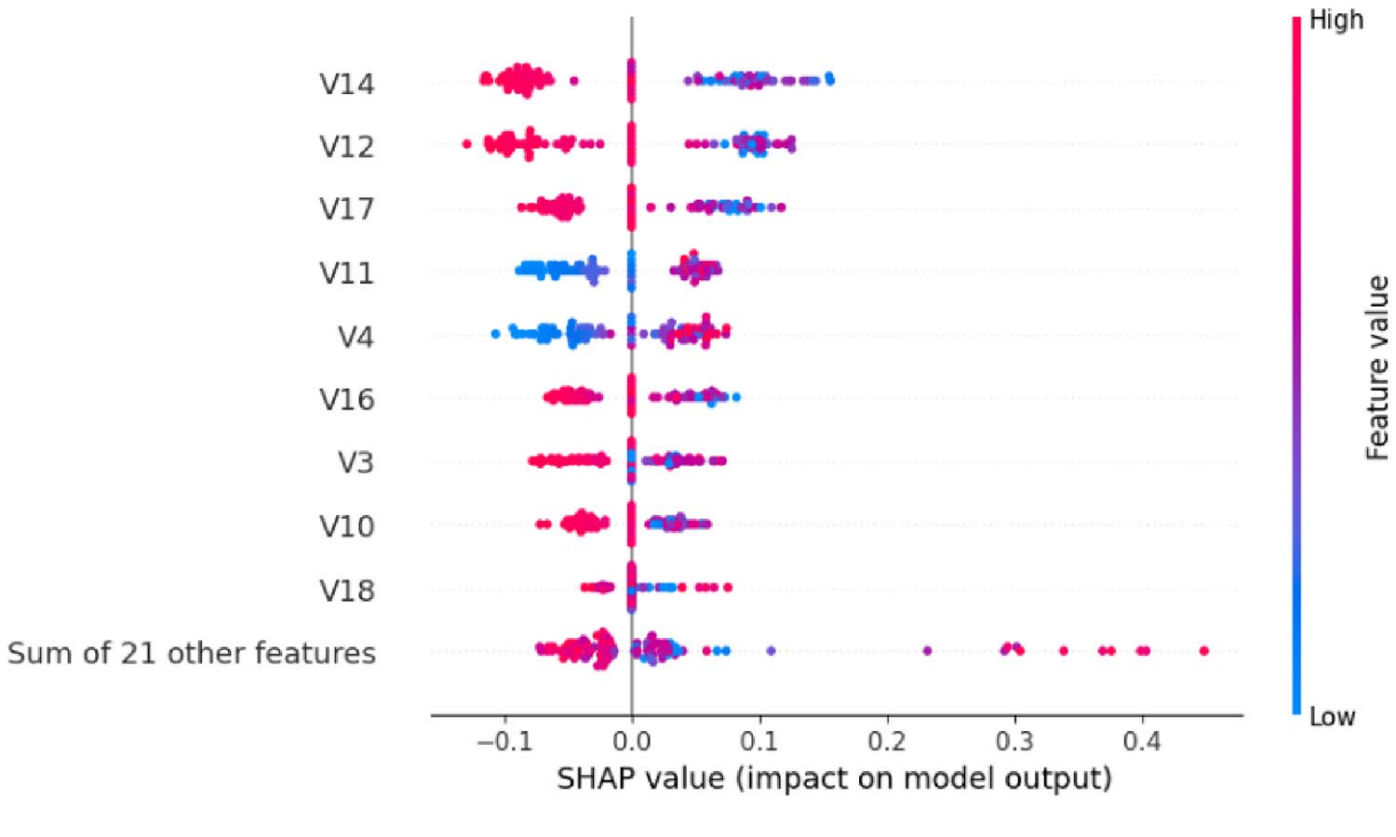
SHAP beeswarm plot for Model 2 showing refined feature impact distributions.

Model 2’s SHAP analysis demonstrates notable improvements in feature utilization efficiency. Figure 18 illustrates the refined impact patterns. The beeswarm plot reveals more concentrated feature impacts, with clearer separation between positive and negative contributions. The feature ordering shows V14, V12, V17, V11, V4, V16, V3, V10, and V18 as the primary contributors.

**Fig. 19:**
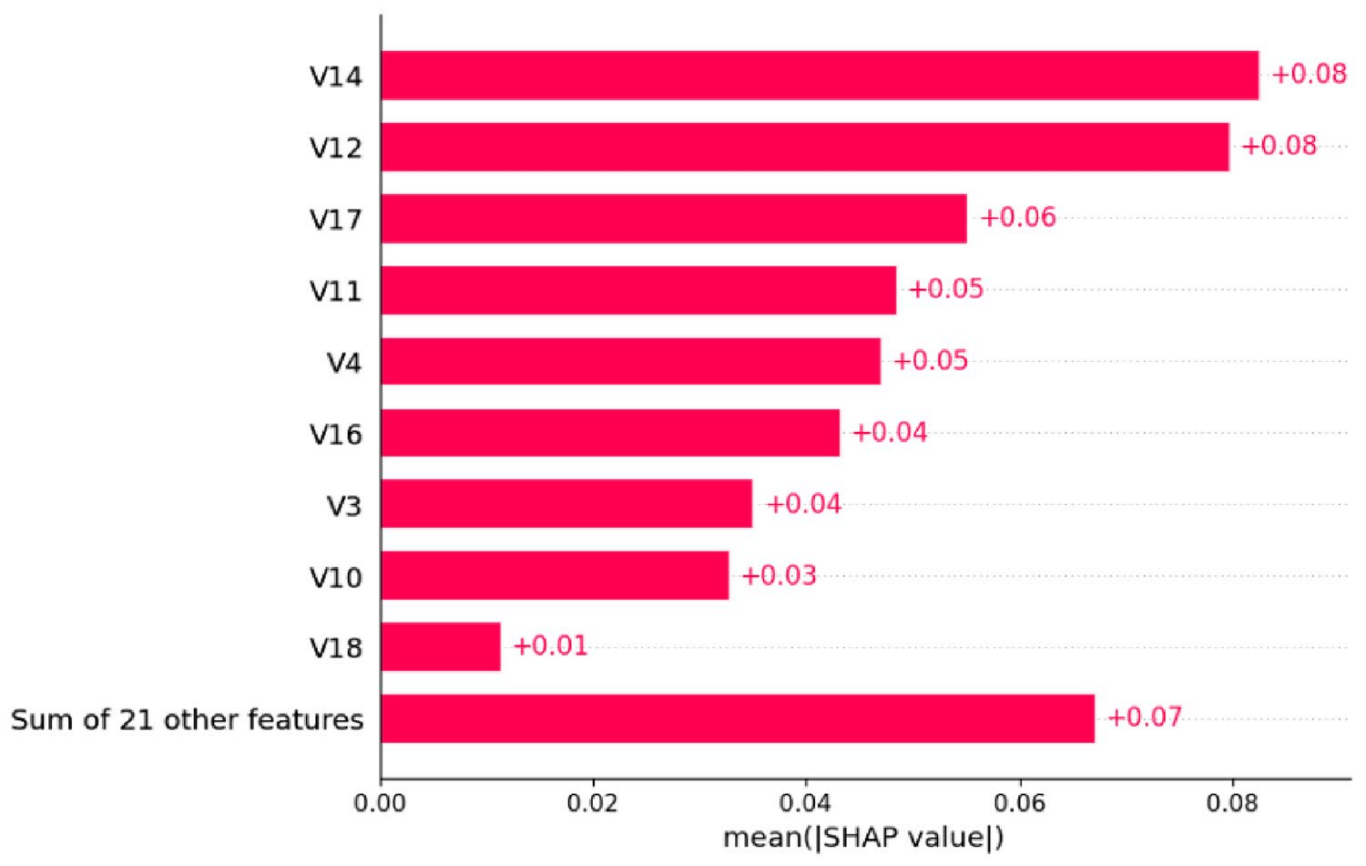
SHAP feature importance ranking for Model 2 with optimized contribution values.

The quantitative feature importance analysis in Fig. 19 confirms the enhanced model performance. Model 2 maintains V14 and V12 as the top performers (0.08 each) while demonstrating improved feature utilization. V17 shows increased importance (0.06 compared to 0.04 in Model 1), and the collective impact of other features is significantly reduced to 0.07, indicating more focused feature utilization.

**Fig. 20:**
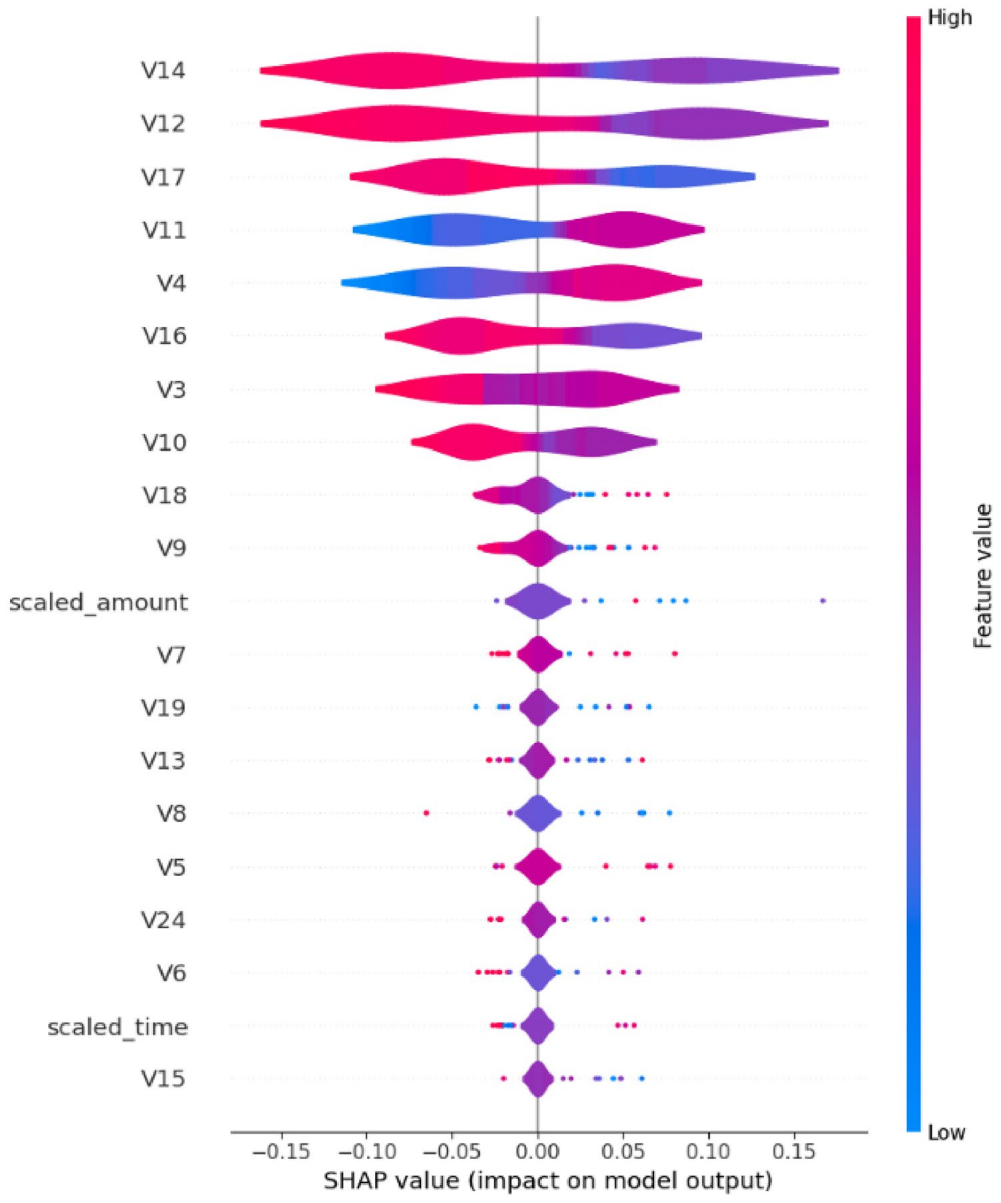
SHAP violin plot for Model 2 showing concentrated feature impact distributions.

Figure 20 presents the distribution analysis. The violin plot demonstrates more concentrated and stable feature contributions, with reduced variability compared to Model 1, suggesting improved model calibration and consistency.

### Comparative model analysis

#### Feature consistency and evolution

The comparative analysis between both models reveals remarkable consistency in critical feature identification. Both models consistently identify V12 and V14 as the primary fraud indicators, with mean SHAP values of 0.08 each across both architectures. This consistency validates the robustness of these features as reliable fraud detection indicators.

The evolution from Model 1 to Model 2 demonstrates several key improvements:

- Enhanced Feature Focus: Model 2 reduces reliance on collective ”other features” from 0.36 to 0.07, indicating more efficient primary feature utilization
- Improved V17 Utilization: V17 shows enhanced importance in Model 2 (0.06) compared to Model 1 (0.04)
- Maintained Core Features: V11, V4, V16, V3, and V10 maintain stable importance rankings across both models
- Refined Distribution Patterns: Model 2 exhibits more concentrated SHAP value distributions, suggesting improved model calibration

#### Explainability validation

The XAI analysis validates both models’ reliability through multiple dimensions:

Consistent Feature Importance: The stability of critical features (V12, V14, V17) across different model architectures builds confidence in their genuine predictive value rather than spurious correlations.

Logical Decision Patterns: SHAP values align with expected fraud detection patterns, where specific transaction characteristics consistently contribute to fraud probability assessments.

Transparent Predictions: The clear visualization of feature contributions through beeswarm plots, importance rankings, and violin plots enables stakeholder understanding and facilitates model validation processes.

### Practical implications and applications

The comprehensive XAI analysis provides several actionable insights for fraud detection system implementation:

Feature Prioritization: Organizations should focus monitoring and feature engineering efforts on V12, V14, and V17, as these features demonstrate consistent high importance across both model architectures.

Model Trustworthiness: The consistent feature importance patterns across models build confidence for production deployment, as the models demonstrate stable and interpretable decision-making processes.

Regulatory Compliance: The transparent decision-making processes revealed through SHAP and LIME analyses support explainability requirements in financial applications, particularly important for regulatory compliance and audit purposes.

System Optimization: Understanding feature contributions enables targeted improvements in data collection pipelines, preprocessing procedures, and real-time monitoring systems.

Risk Assessment Enhancement: The detailed feature impact analysis allows for more nuanced risk scoring and enables financial institutions to better understand the characteristics that drive fraud predictions.

The XAI analysis confirms that both proposed models maintain high interpretability while achieving strong predictive performance, making them suitable candidates for production deployment in credit card fraud detection systems where explainability is paramount.

## Error analysis and model comparison

**Fig. 21:**
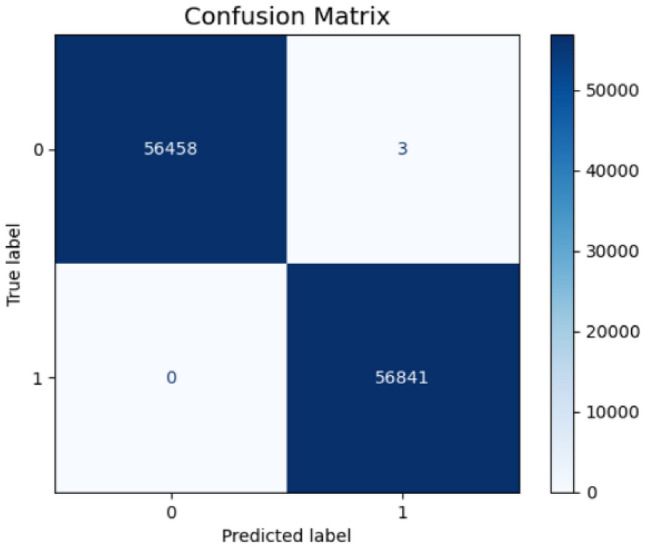
Confusion matrix (Model 1).

**Fig. 22:**
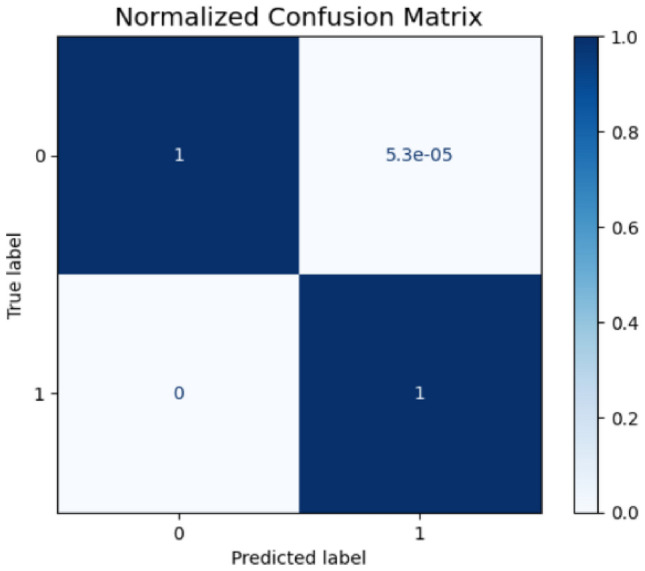
Normalized confusion matrix (Model 1).

**Fig. 23:**
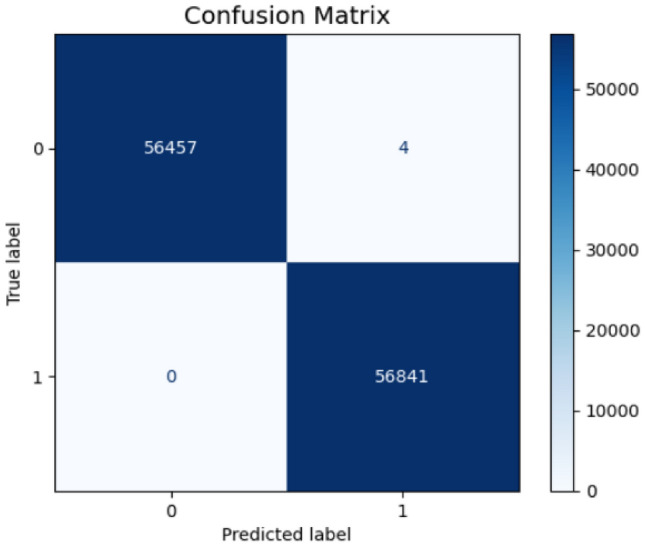
Confusion matrix (Model 2).

**Fig. 24:**
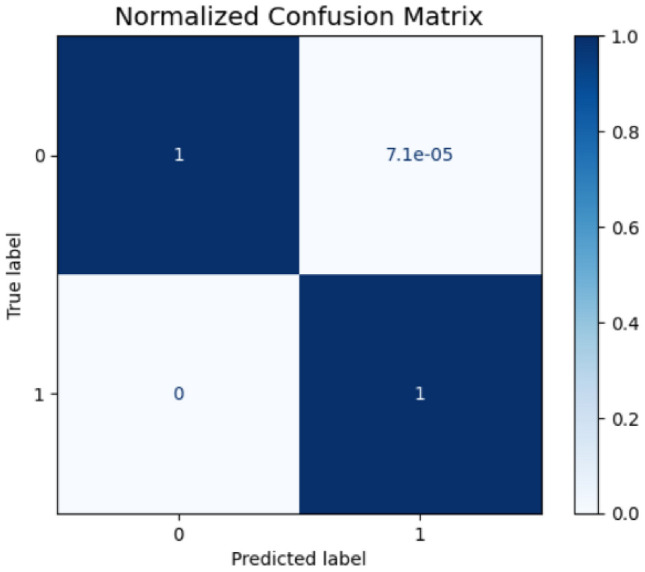
Normalized confusion matrix (Model 2).

The comprehensive error analysis reveals distinct performance characteristics between the two proposed models through detailed examination of confusion matrices, probability distributions, and feature importance patterns. The first model’s confusion matrix (Fig. 21) demonstrates exceptional precision with only 3 false positives out of 56,461 legitimate transactions, achieving a false positive rate of 5.3e-05 as shown in its normalized confusion matrix (Fig. 22). In contrast, the second model’s confusion matrix (Fig. 23) exhibited 4 false positives from 56,457 legitimate cases, resulting in a slightly higher false positive rate of 7.1e-05 (Fig. 24). Both models maintained perfect recall with zero false negatives across 56,841 fraud cases.

**Fig. 25:**
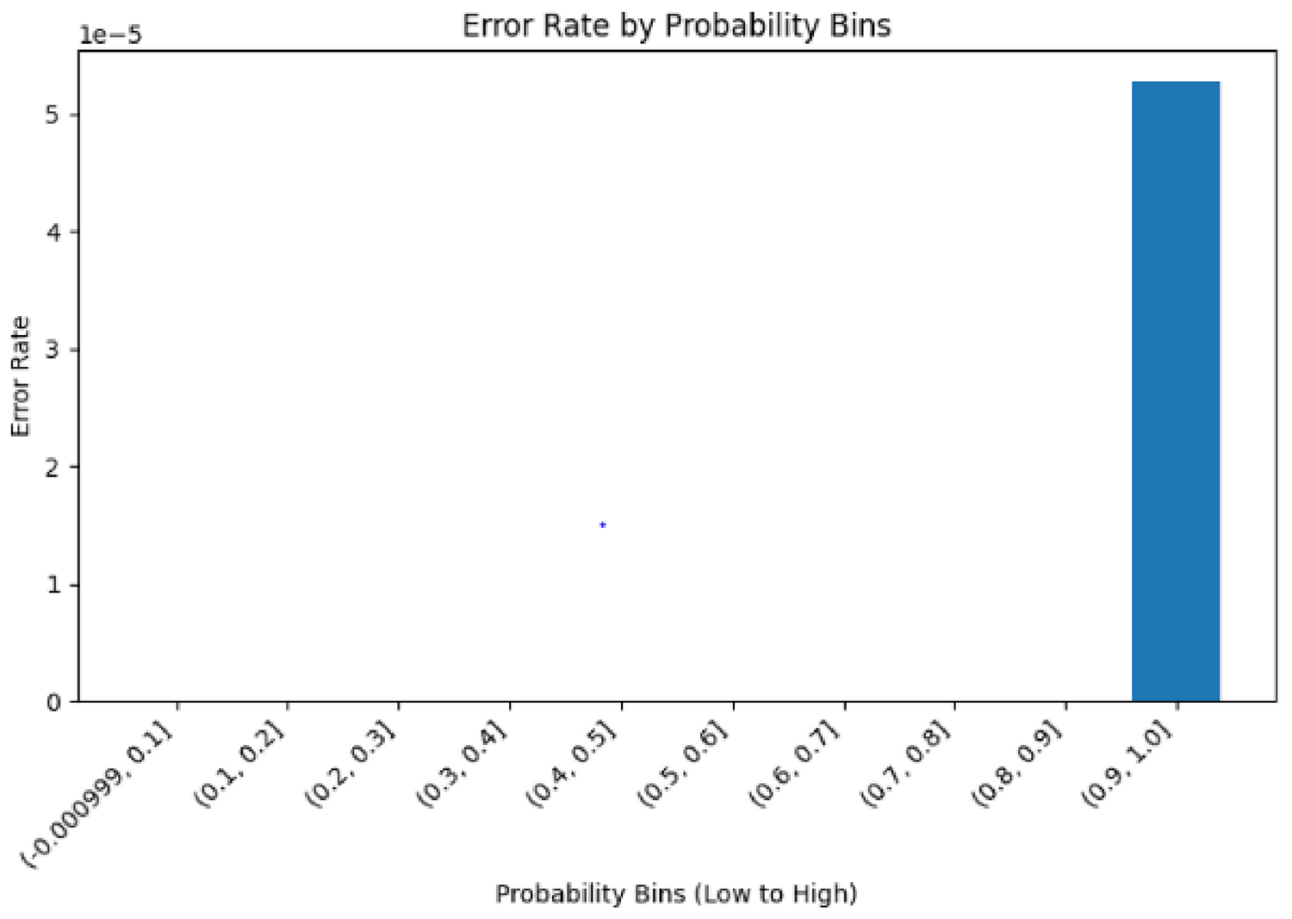
Error rate by bins (Model 1).

**Fig. 26:**
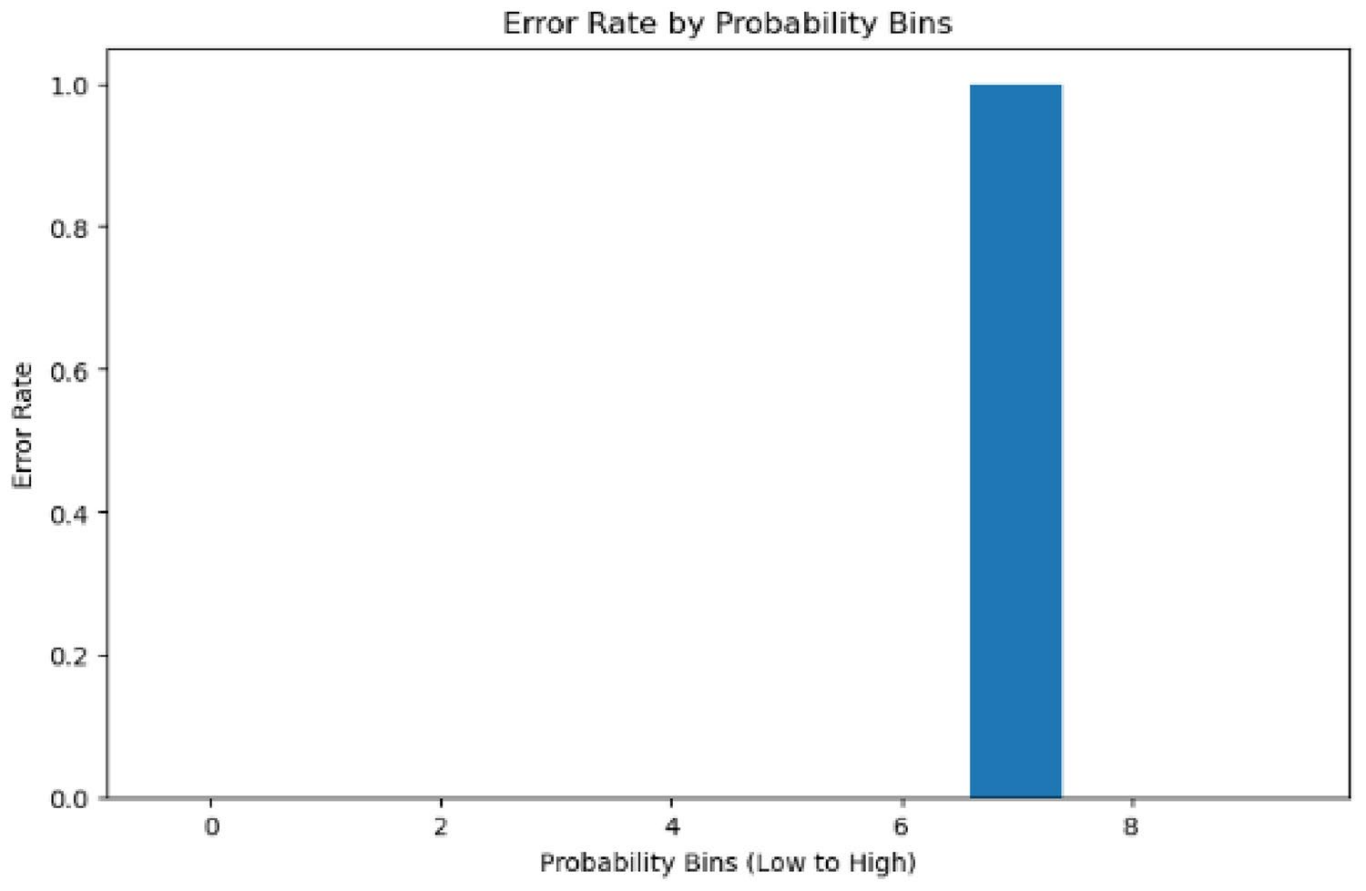
Error rate by bins (Model 2).

The error rate analysis by probability bins reveals critical behavioral differences between models. The first model shows error concentration in specific probability ranges (Fig. 25 with errors occurring predominantly in the highest confidence region (0.9–1.0 range). Conversely, the second model’s error rate by probability bins (Fig. 26) demonstrates a more distributed error pattern, with a notable spike in the 0.6-0.8 confidence range, indicating less consistent confidence calibration.

**Fig. 27:**
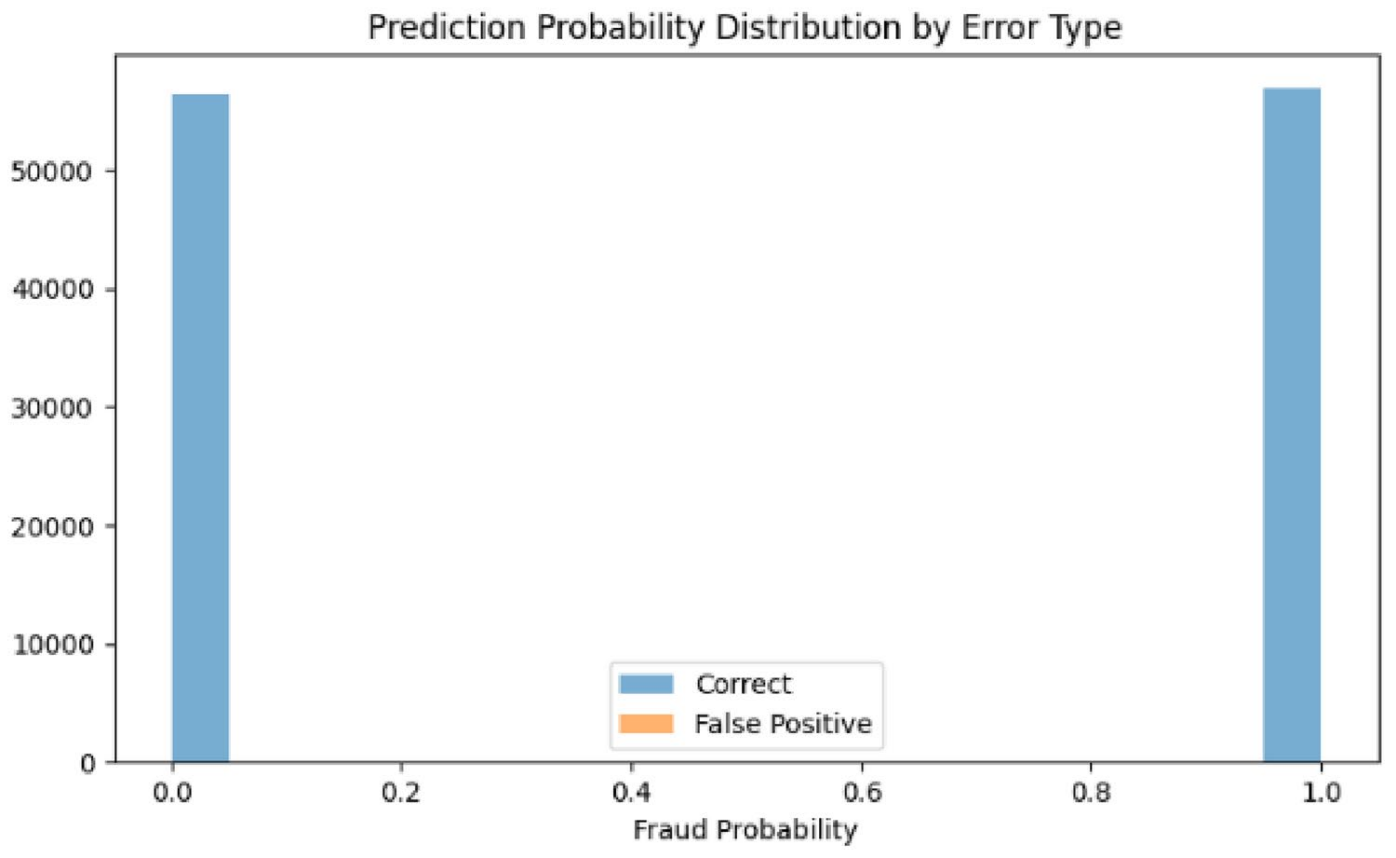
Predicted probability distribution (counts)—Model 1.

**Fig. 28:**
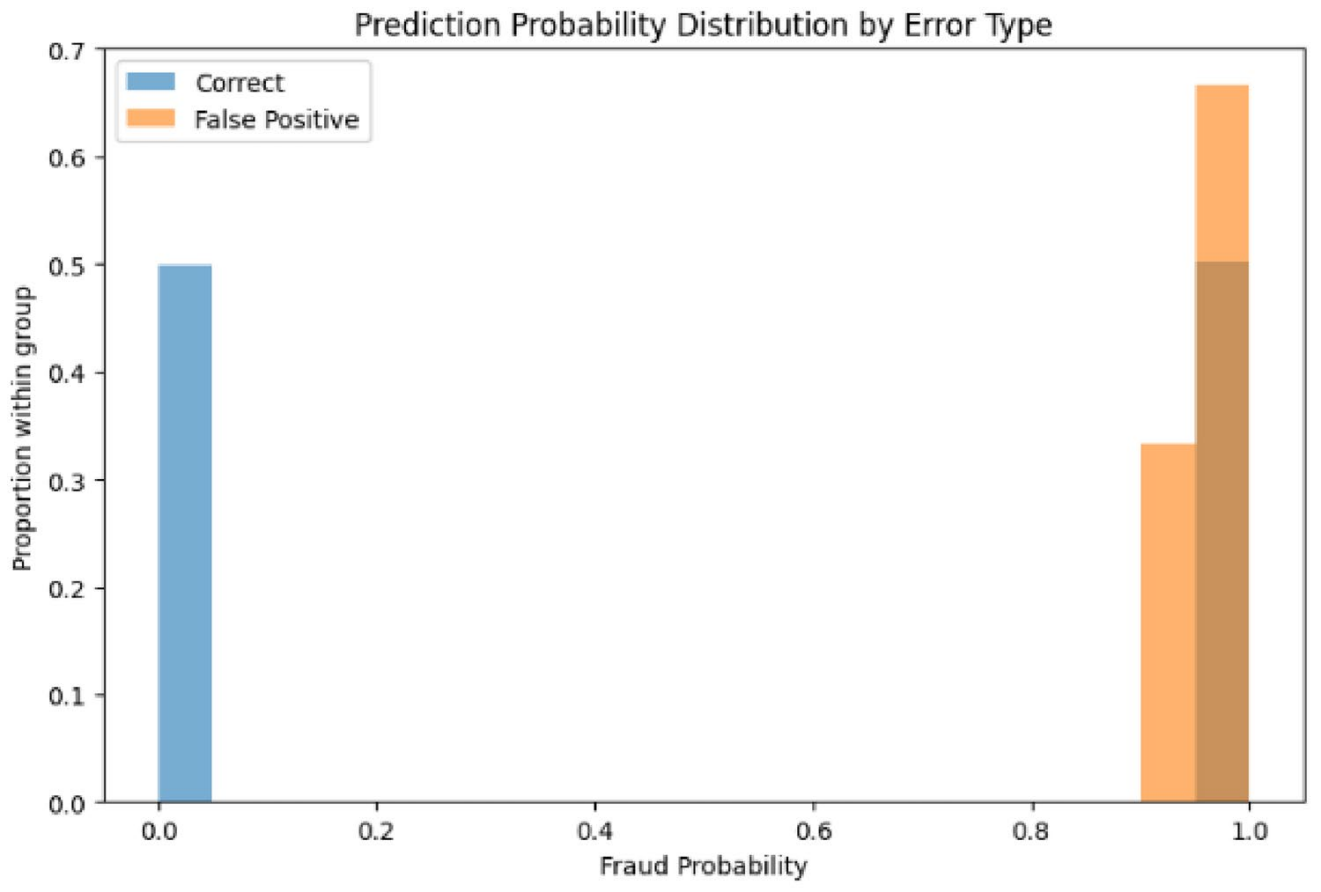
Predicted probability distribution (proportions)—Model 1.

The probability distribution analysis further illuminates model behavior differences. The first model’s probability distributions (Figs. 27 and 28) show false positives concentrated exclusively in the high-confidence region (0.9–1.0), suggesting consistent but occasionally overconfident predictions.

**Fig. 29:**
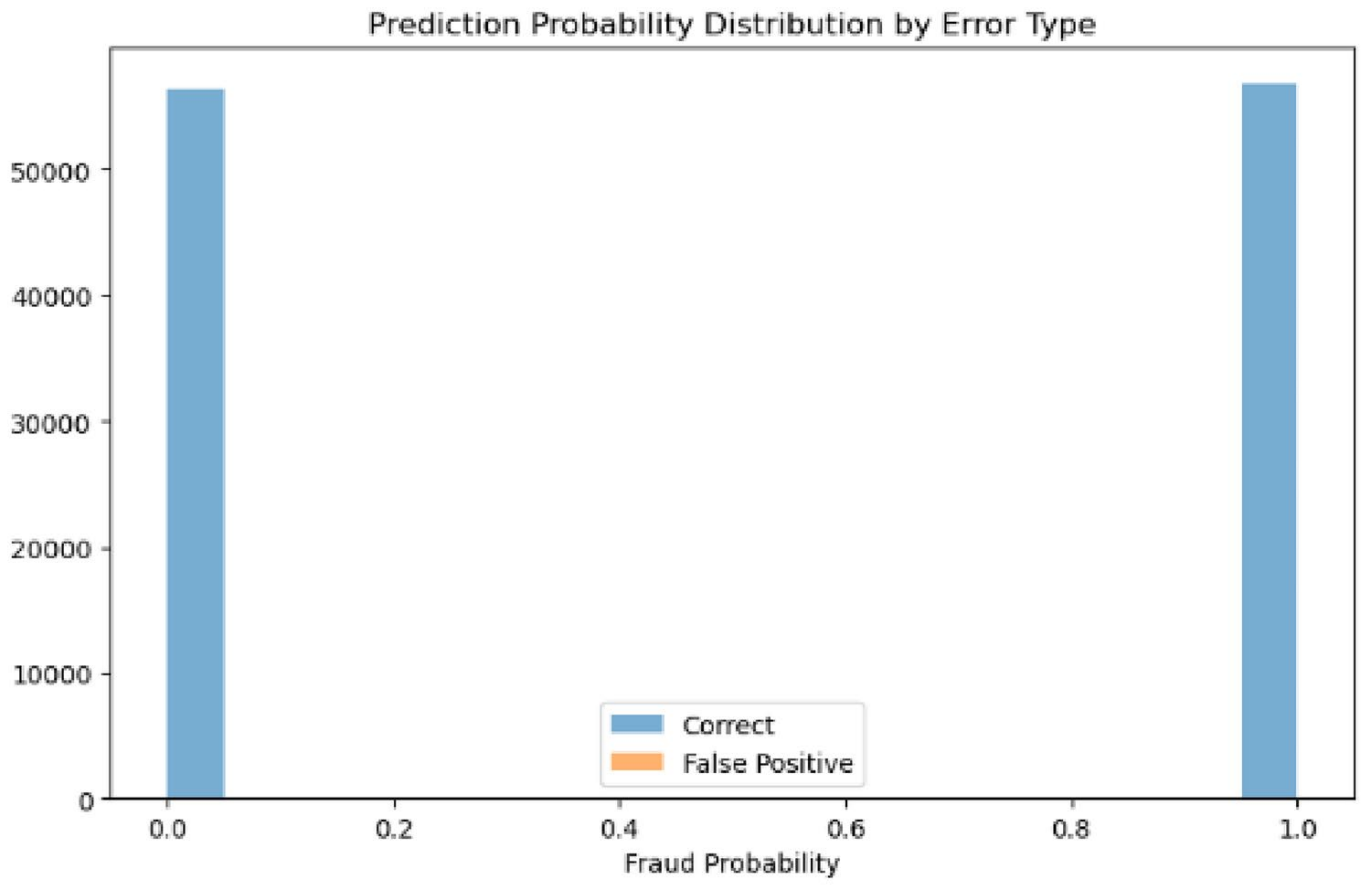
Predicted probability distribution (counts)—Model 2.

**Fig. 30:**
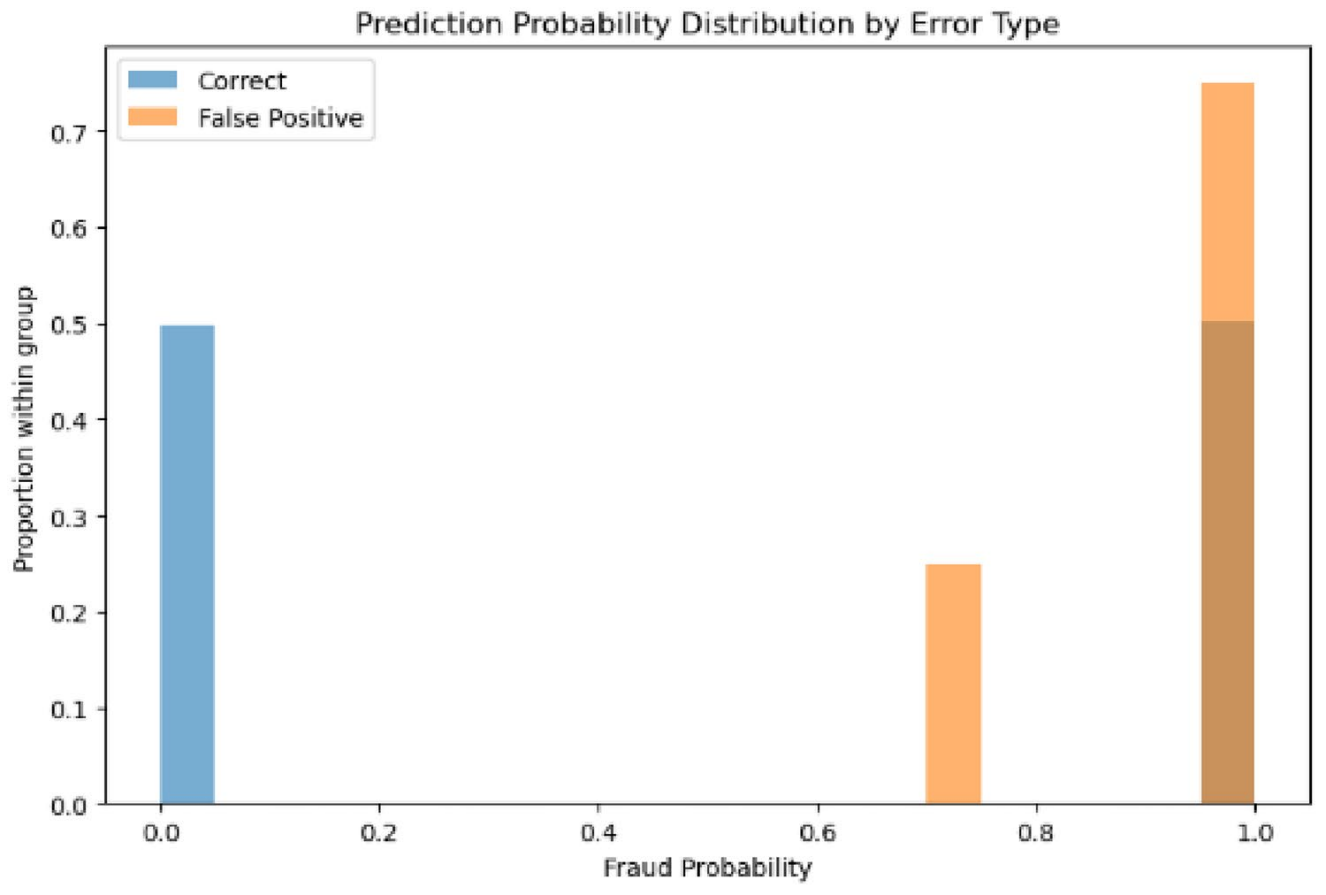
Predicted probability distribution (proportions)—Model 2.

The second model’s probability distributions (Figs. 29 and 30) reveal false positives distributed across multiple confidence levels, with cases appearing in both the 0.6-0.8 and 0.9-1.0 probability ranges, indicating broader confidence intervals and less decisive boundary decisions.

**Fig. 31:**
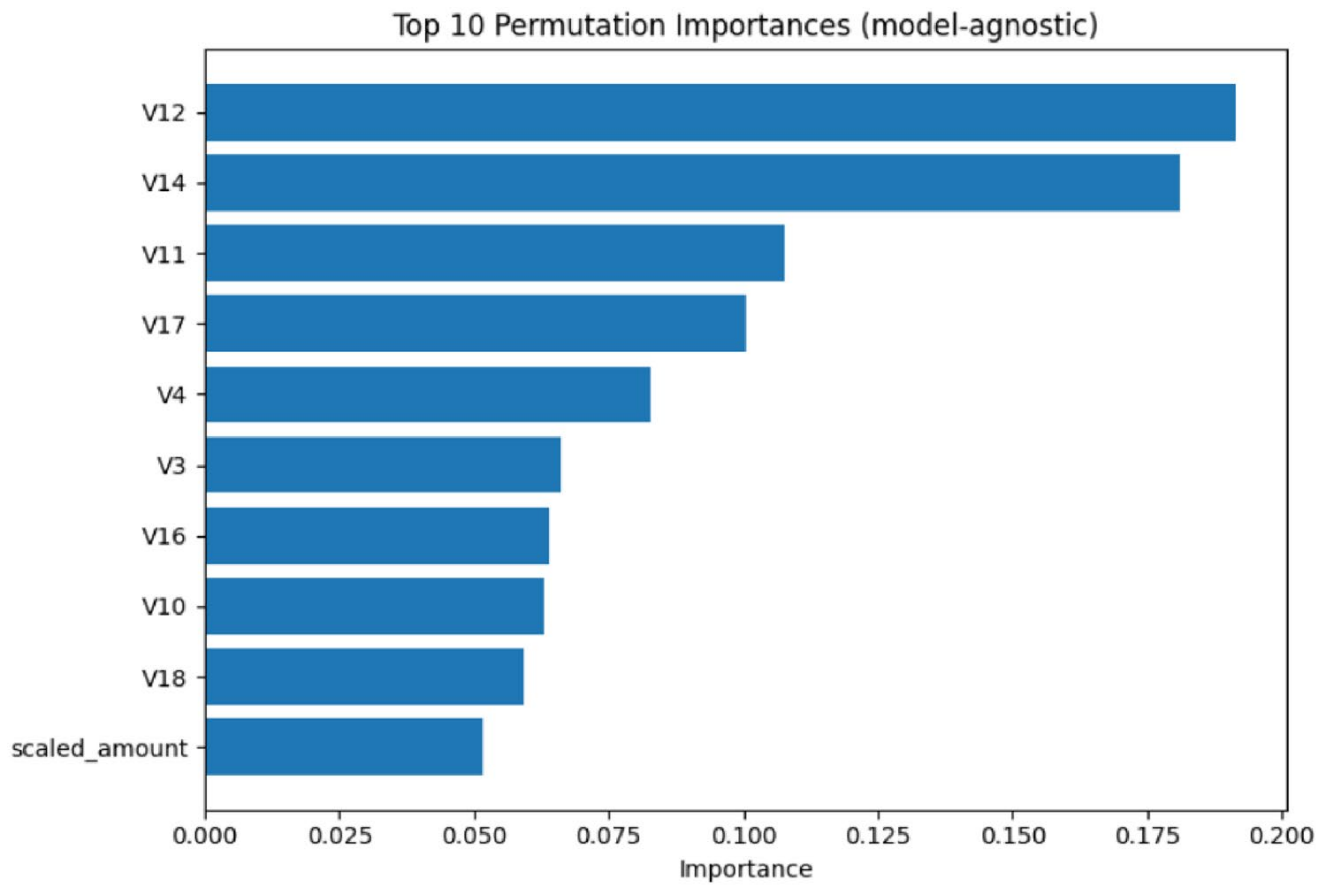
Feature importance (Model 1).

Feature importance analysis demonstrates notable shifts in predictive patterns between models. The first model’s feature importance (Fig. 31) prioritized V12 and V14 as primary features, with V11, V17, and V4 following in importance hierarchy.

**Fig. 32:**
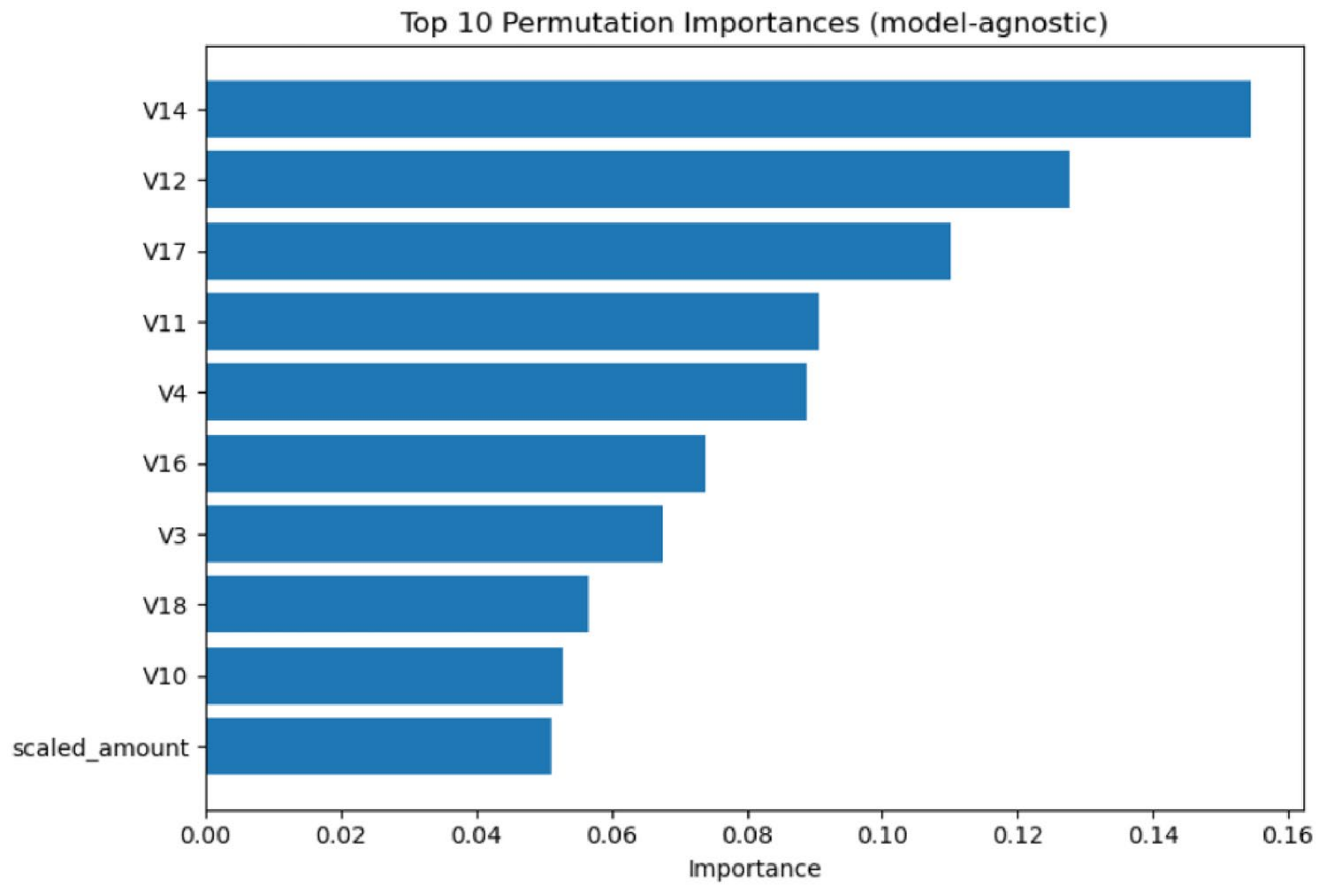
Feature importance (Model 2).

The second model’s feature importance (Fig. 32) elevates V14 as the dominant feature, followed by V12 and V17, showing a reordering of feature priorities. Notably, the scaled_amount feature maintains consistently low importance across both models, reinforcing that transaction value is less predictive than behavioral patterns in fraud detection.

**Fig. 33:**
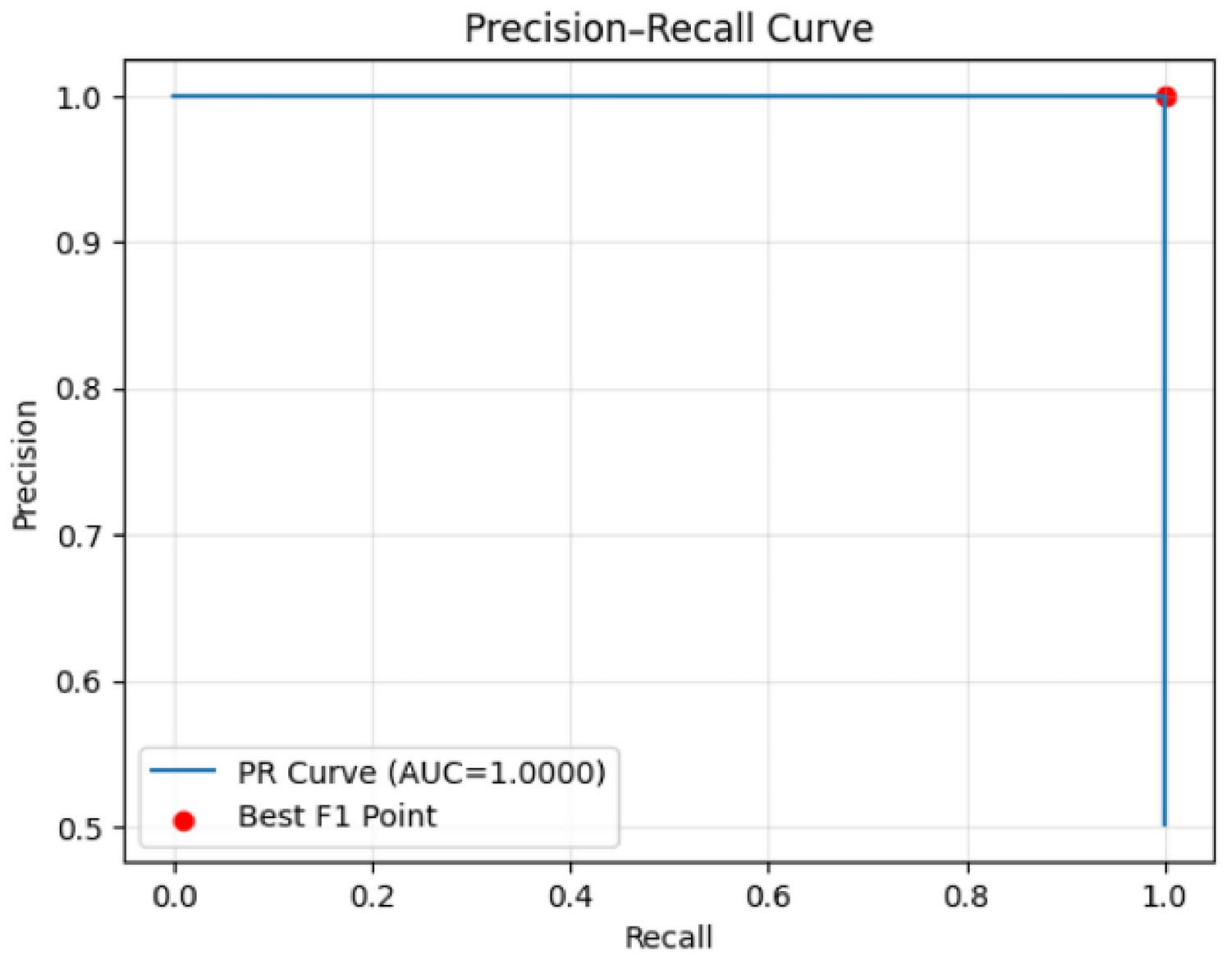
Precision–Recall curve (Model 1).

**Fig. 34:**
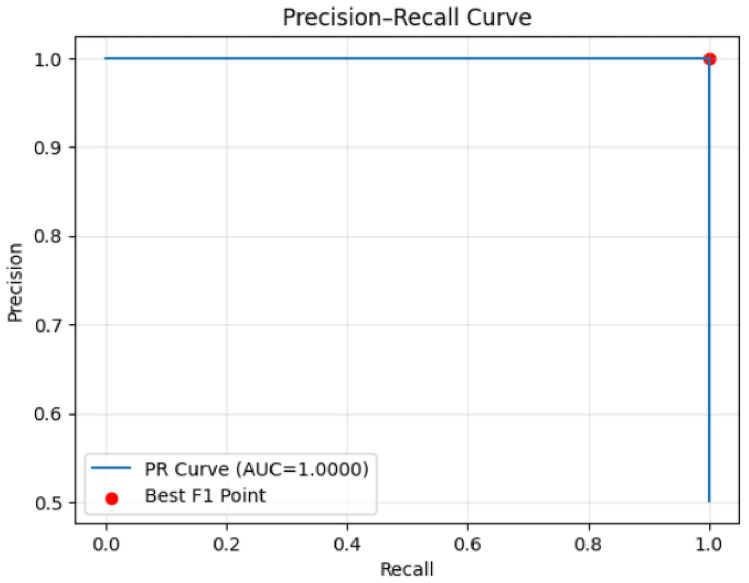
Precision–Recall curve (Model 2).

Both models achieve perfect precision-recall curves with AUC scores of 1.0000 (Figs. 33 and 34), indicating optimal threshold performance across all operating points. However, the distribution of prediction probabilities and error patterns suggests the models operate with different underlying decision boundaries despite similar aggregate performance metrics.

The comprehensive analysis indicates that while both models achieve near-perfect classification performance, the first model demonstrates superior confidence calibration with more consistent error patterns concentrated in high-confidence regions. The second model, while maintaining excellent overall performance, exhibits broader error distribution across confidence levels, potentially indicating less reliable uncertainty estimation. These findings suggest the first model may be more suitable for production deployment where prediction confidence is crucial for downstream decision-making processes and risk management protocols.

## Research limitations

Despite the strong performance of the proposed model, several limitations merit consideration. First, high dimensionality can lead to overfitting, slower training/inference, and reduced interpretability, underscoring the need for careful feature selection or dimensionality reduction. Second, model accuracy depends on effective feature engineering; if salient patterns are not captured, performance may degrade. Third, models trained on historical data may struggle to adapt as fraud strategies evolve over time (concept drift).

In addition, our evaluation relies on synthetic oversampling (e.g., SMOTE/SMOTE-ENN), which can distort class boundaries and increase the risk of overfitting; alternatives such as cost-sensitive losses, focal loss, or generative/anomaly-detection approaches warrant investigation. One of the proposed stacking ensemble is also computationally expensive to train and, in some configurations, to deploy. Finally, scaling to real-time transaction monitoring introduces latency and throughput constraints, as well as continued exposure to drift; a practical deployment may adopt a two-stage pipeline (a fast filter for all transactions followed by the heavier ensemble on flagged cases), streaming/online updates with periodic retraining, and explicit drift detection to sustain performance over time.

## Conclusions and future work

This research focused on improving credit card fraud detection using machine learning, deep learning, and ensemble learning. To handle imbalanced datasets, SMOTE and SMOTE-ENN based on Mahalanobis distance were tested, with SMOTE consistently outperforming SMOTE-ENN across all evaluation metrics. When combined with the proposed ensemble model, SMOTE further improved performance. Among the 37 baseline classifiers and 2 proposed stacking ensemble models, the proposed ensembles demonstrated superior performance. The first model integrates Extra Trees (ET), Convolutional Neural Networks (CNN), Long Short-Term Memory (LSTM), and XGBoost as a meta-learner. The second model combines ET, AdaBoost (with ET and Random Forest as base learners), and XGBoost as the meta-learner. Both models showed outstanding performance.

Experiments using European credit card transaction data confirmed the models’ robustness and adaptability, achieving near-perfect metrics: accuracy, precision, recall, F1 score, and AUC of up to 1.0. These results highlight their potential for real-world deployment.

### Deployment guidance

Use a two-step setup: a fast model scores every transaction, and the heavier ensemble runs only on the flagged ones. Set the alert threshold based on your fraud/false-alarm costs and refresh it with recent data. Track precision and recall, watch for data changes, and retrain on a schedule or when performance drops. Give simple reasons for each alert and keep audit logs. Keep latency low with a smaller/distilled model and autoscaling. Roll out in stages before full production.

Future research could explore adding more machine and deep learning models or applying this ensemble strategy to other financial fraud types. Investigating real-time detection and scalability for large datasets would further validate its practicality. As fraud tactics evolve, ongoing innovation is crucial, and this ensemble framework offers a strong foundation for future advancements. In addition, addressing concept drift—the natural evolution of fraud patterns over time – will be essential. Future studies may investigate strategies such as periodic retraining, drift detection, and online updating to ensure long-term adaptability and robustness.

## Data Availability

The dataset provided during the current study are available : Credit Card Dataset.

## Code availability

The complete source code is available for review at Source Code.
